# Prospective validation of ORACLE, a clonal expression biomarker associated with survival of patients with lung adenocarcinoma

**DOI:** 10.1038/s43018-024-00883-1

**Published:** 2025-01-09

**Authors:** Dhruva Biswas, Yun-Hsin Liu, Javier Herrero, Yin Wu, David A. Moore, Takahiro Karasaki, Kristiana Grigoriadis, Wei-Ting Lu, Selvaraju Veeriah, Cristina Naceur-Lombardelli, Neil Magno, Sophia Ward, Alexander M. Frankell, Mark S. Hill, Emma Colliver, Sophie de Carné Trécesson, Philip East, Aman Malhi, Daniel M. Snell, Olga O’Neill, Daniel Leonce, Johanna Mattsson, Amanda Lindberg, Patrick Micke, Judit Moldvay, Zsolt Megyesfalvi, Balazs Dome, János Fillinger, Jerome Nicod, Julian Downward, Zoltan Szallasi, TRACERx Consortium, Dhruva Biswas, Yin Wu, David A. Moore, Takahiro Karasaki, Kristiana Grigoriadis, Wei-Ting Lu, Selvaraju Veeriah, Cristina Naceur-Lombardelli, Sophia Ward, Alexander M. Frankell, Emma Colliver, Jerome Nicod, Zoltan Szallasi, Ariana Huebner, Corentin Richard, Crispin T. Hiley, Emilia L. Lim, Francisco Gimeno-Valiente, Krupa Thakkar, Maise Al Bakir, Monica Sivakumar, Ieva Usaite, Sadegh Saghafinia, Sharon Vanloo, Sian Harries, Antonia Toncheva, Paulina Prymas, Bushra Mussa, Michalina Magala, Elizabeth Keene, Abigail Bunkum, Carlos Martínez-Ruiz, Clare Puttick, Despoina Karagianni, James R. M. Black, Kerstin Thol, Nicholas McGranahan, Olivia Lucas, Robert Bentham, Roberto Vendramin, Sergio A. Quezada, Simone Zaccaria, Sonya Hessey, Supreet Kaur Bola, Wing Kin Liu, Rija Zaidi, Lucrezia Patruno, Martin D. Forster, Siow Ming Lee, Gareth A. Wilson, Rachel Rosenthal, Andrew Rowan, Chris Bailey, Claudia Lee, Katey S. S. Enfield, Mihaela Angelova, Oriol Pich, Cian Murphy, Maria Zagorulya, Michelle M. Leung, Teresa Marafioti, Elaine Borg, Mary Falzon, Reena Khiroya, Thomas Patrick Jones, Sarah Benafif, Dionysis Papadatos-Pastos, James Wilson, Tanya Ahmad, Angela Dwornik, Angeliki Karamani, Benny Chain, David R. Pearce, Georgia Stavrou, Gerasimos-Theodoros Mastrokalos, Helen L. Lowe, James L. Reading, John A. Hartley, Kayalvizhi Selvaraju, Leah Ensell, Mansi Shah, Maria Litovchenko, Piotr Pawlik, Samuel Gamble, Seng Kuong Anakin Ung, Victoria Spanswick, Clare E. Weeden, Eva Grönroos, Jacki Goldman, Mickael Escudero, Philip Hobson, Stefan Boeing, Tamara Denner, Vittorio Barbè, William Hill, Yutaka Naito, Erik Sahai, Zoe Ramsden, George Kassiotis, Imran Noorani, Jason F. Lester, Amrita Bajaj, Apostolos Nakas, Azmina Sodha-Ramdeen, Mohamad Tufail, Molly Scotland, Rebecca Boyles, Sridhar Rathinam, Sean Dulloo, Dean A. Fennell, Claire Wilson, Gurdeep Matharu, Jacqui A. Shaw, Ekaterini Boleti, Heather Cheyne, Mohammed Khalil, Shirley Richardson, Tracey Cruickshank, Gillian Price, Keith M. Kerr, Jack French, Kayleigh Gilbert, Babu Naidu, Akshay J. Patel, Aya Osman, Mandeesh Sangha, Gerald Langman, Helen Shackleford, Madava Djearaman, Gary Middleton, Angela Leek, Jack Davies Hodgkinson, Nicola Totton, Eustace Fontaine, Felice Granato, Juliette Novasio, Kendadai Rammohan, Leena Joseph, Paul Bishop, Vijay Joshi, Sara Waplington, Adam Atkin, Antonio Paiva-Correia, Philip Crosbie, Katherine D. Brown, Mathew Carter, Anshuman Chaturvedi, Pedro Oliveira, Colin R. Lindsay, Fiona H. Blackhall, Yvonne Summers, Matthew G. Krebs, Jonathan Tugwood, Caroline Dive, Hugo J. W. L. Aerts, Roland F. Schwarz, Tom L. Kaufmann, Peter Van Loo, Carla Castignani, Roberto Salgado, Miklos Diossy, Jonas Demeulemeester, Stephan Beck, Emma Nye, Richard Kevin Stone, Jayant K. Rane, Jeanette Kittel, Kerstin Haase, Kexin Koh, Rachel Scott, Karl S. Peggs, Emilie Martinoni Hoogenboom, Fleur Monk, James W. Holding, Junaid Choudhary, Kunal Bhakhri, Pat Gorman, Robert C. M. Stephens, Yien Ning Sophia Wong, Maria Chiara Pisciella, Steve Bandula, Thomas B. K. Watkins, Catarina Veiga, Gary Royle, Charles-Antoine Collins-Fekete, Francesco Fraioli, Paul Ashford, Alexander James Procter, Asia Ahmed, Magali N. Taylor, Arjun Nair, David Lawrence, Davide Patrini, Neal Navani, Ricky M. Thakrar, Sam M. Janes, Zoltan Kaplar, Allan Hackshaw, Camilla Pilotti, Rachel Leslie, Anne-Marie Hacker, Sean Smith, Aoife Walker, Anca Grapa, Hanyun Zhang, Khalid AbdulJabbar, Xiaoxi Pan, Yinyin Yuan, David Chuter, Mairead MacKenzie, Serena Chee, Patricia Georg, Aiman Alzetani, Judith Cave, Eric Lim, Paulo De Sousa, Simon Jordan, Alexandra Rice, Hilgardt Raubenheimer, Harshil Bhayani, Lyn Ambrose, Anand Devaraj, Hemangi Chavan, Sofina Begum, Silviu I. Buderi, Daniel Kaniu, Mpho Malima, Sarah Booth, Andrew G. Nicholson, Nadia Fernandes, Pratibha Shah, Chiara Proli, Madeleine Hewish, Sarah Danson, Michael J. Shackcloth, Lily Robinson, Peter Russell, Kevin G. Blyth, Andrew Kidd, Craig Dick, John Le Quesne, Alan Kirk, Mo Asif, Rocco Bilancia, Nikos Kostoulas, Jennifer Whiteley, Mathew Thomas, Mariam Jamal-Hanjani, Nnennaya Kanu, Nicolai J. Birkbak, Charles Swanton, Allan Hackshaw, Mariam Jamal-Hanjani, Nnennaya Kanu, Nicolai J. Birkbak, Charles Swanton

**Affiliations:** 1 Cancer Research UK Lung Cancer Centre of Excellence, University College London Cancer Institute, London, UK; 2 Bill Lyons Informatics Centre, University College London Cancer Institute, London, UK; 3 Cancer Evolution and Genome Instability Laboratory, The Francis Crick Institute, London, UK; 4 Centre for Inflammation Biology and Cancer Immunology, King’s College London, London, UK; 5 Department of Medical Oncology, Guy’s Hospital, London, UK; 6 Department of Cellular Pathology, University College London Hospitals, London, UK; 7 Cancer Metastasis Lab, University College London Cancer Institute, London, UK; 8 Department of Thoracic Surgery, Respiratory Center, Toranomon Hospital, Tokyo, Japan; 9 Cancer Genome Evolution Research Group, Cancer Research UK Lung Cancer Centre of Excellence, University College London Cancer Institute, London, UK; 10 Genomics Science Technology Platform, The Francis Crick Institute, London, UK; 11 Oncogene Biology Laboratory, The Francis Crick Institute, London, UK; 12 Bioinformatics and Biostatistics, The Francis Crick Institute, London, UK; 13 Cancer Research UK and University College London Cancer Trials Centre, University College London, London, UK; 14 Department of Immunology, Genetics and Pathology, Uppsala University, Uppsala, Sweden; 15 1st Department of Pulmonology, National Koranyi Institute of Pulmonology, Budapest, Hungary; 16 Department of Pulmonology, University of Szeged Albert Szent-Gyorgyi Medical School, Szeged, Hungary; 17 National Koranyi Institute of Pulmonology, Budapest, Hungary; 18 Department of Thoracic Surgery, Semmelweis University and National Institute of Oncology, Budapest, Hungary; 19 Department of Thoracic Surgery, Comprehensive Cancer Center, Medical University of Vienna, Vienna, Austria; 20 Department of Translational Medicine, Lund University, Lund, Sweden; 21 Computational Health Informatics Program, Boston Children’s Hospital, Harvard Medical School, Boston, MA USA; 22 Department of Oncology, University College London Hospitals, London, UK; 23 Department of Molecular Medicine, Aarhus University Hospital, Aarhus, Denmark; 24 Department of Clinical Medicine, Aarhus University, Aarhus, Denmark; 25 Computational Cancer Genomics Research Group, University College London Cancer Institute, London, UK; 26 Immune Regulation and Tumour Immunotherapy Group, Cancer Immunology Unit, Research Department of Haematology, University College London Cancer Institute, London, UK; 27 Cancer Genome Evolution Research Group, University College London Cancer Institute, London, UK; 28 University College London Hospitals, London, UK; 29 Tumour Immunogenomics and Immunosurveillance Laboratory, University College London Cancer Institute, London, UK; 30 Cancer Research UK Lung Cancer Centre of Excellence, University College London, Cancer Institute, London, UK; 31 The Whittington Hospital NHS Trust, London, UK; 32 University College London Cancer Institute, London, UK; 33 The Francis Crick Institute, London, UK; 34 Department of Infectious Disease, Faculty of Medicine, Imperial College London, London, UK; 35 Department of Neurosurgery, National Hospital for Neurology and Neurosurgery, London, UK; 36 University College London, London, UK; 37 Singleton Hospital, Swansea Bay University Health Board, Swansea, UK; 38 University Hospitals of Leicester NHS Trust, Leicester, UK; 39 University of Leicester, Leicester, UK; 40 Leicester Medical School, University of Leicester, Leicester, UK; 41 Cancer Research Centre, University of Leicester, Leicester, UK; 42 Royal Free London NHS Foundation Trust, London, UK; 43 Aberdeen Royal Infirmary NHS Grampian, Aberdeen, UK; 44 Department of Medical Oncology, Aberdeen Royal Infirmary NHS Grampian, Aberdeen, UK; 45 University of Aberdeen, Aberdeen, UK; 46 Department of Pathology, Aberdeen Royal Infirmary NHS Grampian, Aberdeen, UK; 47 Birmingham Acute Care Research Group, Institute of Inflammation and Ageing, University of Birmingham, Birmingham, UK; 48 Guy’s and St Thomas’ NHS Foundation Trust, London, UK; 49 University Hospital Birmingham NHS Foundation Trust, Birmingham, UK; 50 Institute of Immunology and Immunotherapy, University of Birmingham, Birmingham, UK; 51 Manchester Cancer Research Centre Biobank, Manchester, UK; 52 Wythenshawe Hospital, Manchester University NHS Foundation Trust, Wythenshawe, UK; 53 Manchester University NHS Foundation Trust, Manchester, UK; 54 Wythenshawe Hospital, Manchester University NHS Foundation Trust, Manchester, UK; 55 Division of Infection, Immunity and Respiratory Medicine, University of Manchester, Manchester, UK; 56 Cancer Research UK Lung Cancer Centre of Excellence, University of Manchester, Manchester, UK; 57 The Christie NHS Foundation Trust, Manchester, UK; 58 Division of Cancer Sciences, The University of Manchester and The Christie NHS Foundation Trust, Manchester, UK; 59 CRUK Manchester Institute Cancer Biomarker Centre, University of Manchester, Manchester, UK; 60 CRUK Lung Cancer Centre of Excellence, University of Manchester, Manchester, UK; 61 Artificial Intelligence in MedicineAIM Program, Mass General Brigham, Harvard Medical School, Boston, MA USA; 62 Department of Radiation Oncology, Brigham and Women’s Hospital, Dana-Farber Cancer Institute, Harvard Medical School, Boston, MA USA; 63 Radiology and Nuclear Medicine, CARIM and GROW, Maastricht University, Maastricht, The Netherlands; 64 Institute for Computational Cancer Biology, Center for Integrated OncologyCIO, Cancer Research Center Cologne EssenCCCE, Faculty of Medicine and University Hospital Cologne, University of Cologne, Cologne, Germany; 65 Berlin Institute for the Foundations of Learning and DataBIFOLD, Berlin, Germany; 66 Berlin Institute for Medical Systems Biology, Max Delbrück Center for Molecular Medicine in the Helmholtz Association, Berlin, Germany; 67 Department of Genetics, The University of Texas MD Anderson Cancer Center, Houston, TX USA; 68 Department of Genomic Medicine, The University of Texas MD Anderson Cancer Center, Houston, TX USA; 69 Cancer Genomics Laboratory, The Francis Crick Institute, London, UK; 70 Medical Genomics, University College London Cancer Institute, London, UK; 71 Department of Pathology, ZAS Hospitals, Antwerp, Belgium; 72 Division of Research, Peter MacCallum Cancer Centre, Melbourne, Australia; 73 Danish Cancer Institute, Copenhagen, Denmark; 74 Computational Health Informatics Program, Boston Children’s Hospital, Boston, MA USA; 75 Department of Physics of Complex Systems, ELTE Eötvös Loránd University, Budapest, Hungary; 76 Integrative Cancer Genomics Laboratory, VIB Center for Cancer Biology, Leuven, Belgium; 77 VIB Center for AI & Computational Biology, Leuven, Belgium; 78 Department of Oncology, KU Leuven, Leuven, Belgium; 79 Experimental Histopathology, The Francis Crick Institute, London, UK; 80 University College London Cancer Institute, London, UK and Cancer Evolution and Genome Instability Laboratory, The Francis Crick Institute, London, UK; 81 Cancer Metastasis Laboratory, University College London Cancer Institute, London, UK; 82 Cancer Research UK Lung Cancer Centre of Excellence, UCL Cancer Institute, London, UK; 83 Department of Haematology, University College London Hospitals, London, UK; 84 Cancer Immunology Unit, Research Department of Haematology, University College London Cancer Institute, London, UK; 85 National Cancer Centre, Singapore, Singapore; 86 Department of Pathology, Stanford University School of Medicine, Stanford, CA USA; 87 Centre for Medical Image Computing, Department of Medical Physics and Biomedical Engineering, London, UK; 88 Department of Medical Physics and Bioengineering, University College London Cancer Institute, London, UK; 89 Department of Medical Physics and Biomedical Engineering, University College London, London, UK; 90 Institute of Nuclear Medicine, Division of Medicine, University College London, London, UK; 91 Institute of Structural and Molecular Biology, University College London, London, UK; 92 Department of Radiology, University College London Hospitals, London, UK; 93 UCL Respiratory, Department of Medicine, University College London, London, UK; 94 Department of Thoracic Surgery, University College London Hospital NHS Trust, London, UK; 95 Lungs for Living Research Centre, UCL Respiratory, University College London, London, UK; 96 Department of Thoracic Medicine, University College London Hospitals, London, UK; 97 Lungs for Living Research Centre, UCL Respiratory, Department of Medicine, University College London, London, UK; 98 Integrated Radiology Department, North-Buda St John’s Central Hospital, Budapest, Hungary; 99 Institute of Nuclear Medicine, University College London Hospitals, London, UK; 100 Cancer Research UK and UCL Cancer Trials Centre, London, UK; 101 The Institute of Cancer Research, London, UK; 102 Garvan Institute of Medical Research, Sydeny, New South Wales Australia; 103 Case45, London, UK; 104 The University of Texas MD Anderson Cancer Center, Houston, TX USA; 105 The University of Texas MD Anderson Cancer Center, Houston, USA; 106 Independent Cancer Patient’s voice, London, UK; 107 University Hospital Southampton NHS Foundation Trust, Southampton, UK; 108 The NIHR Southampton Biomedical Research Centre, University Hospital Southampton NHS Foundation Trust, Southampton, UK; 109 Department of Oncology, University Hospital Southampton NHS Foundation Trust, Southampton, UK; 110 Academic Division of Thoracic Surgery, Imperial College London, London, UK; 111 Royal Brompton and Harefield Hospitals, part of Guy’s and St Thomas’ NHS Foundation Trust, London, UK; 112 National Heart and Lung Institute, Imperial College, London, UK; 113 Royal Surrey Hospital, Royal Surrey Hospitals NHS Foundation Trust, Guildford, UK; 114 University of Surrey, Guildford, UK; 115 University of Sheffield, Sheffield, UK; 116 Sheffield Teaching Hospitals NHS Foundation Trust, Sheffield, UK; 117 Liverpool Heart and Chest Hospital, Liverpool, UK; 118 Princess Alexandra Hospital, The Princess Alexandra Hospital NHS Trust, Harlow, UK; 119 School of Cancer Sciences, University of Glasgow, Glasgow, UK; 120 Beatson Institute for Cancer Research, University of Glasgow, Glasgow, UK; 121 Queen Elizabeth University Hospital, Glasgow, UK; 122 Institute of Infection, Immunity and Inflammation, University of Glasgow, Glasgow, UK; 123 NHS Greater Glasgow and Clyde, Glasgow, UK; 124 Cancer Research UK Scotland Institute, Glasgow, UK; 125 Institute of Cancer Sciences, University of Glasgow, Glasgow, UK; 126 NHS Greater Glasgow and Clyde Pathology Department, Queen Elizabeth University Hospital, Glasgow, UK; 127 Golden Jubilee National Hospital, Clydebank, UK

**Keywords:** Tumour biomarkers, Non-small-cell lung cancer, Cancer genomics, Cancer

## Abstract

Human tumors are diverse in their natural history and response to treatment, which in part results from genetic and transcriptomic heterogeneity. In clinical practice, single-site needle biopsies are used to sample this diversity, but cancer biomarkers may be confounded by spatiogenomic heterogeneity within individual tumors. Here we investigate clonally expressed genes as a solution to the sampling bias problem by analyzing multiregion whole-exome and RNA sequencing data for 450 tumor regions from 184 patients with lung adenocarcinoma in the TRACERx study. We prospectively validate the survival association of a clonal expression biomarker, Outcome Risk Associated Clonal Lung Expression (ORACLE), in combination with clinicopathological risk factors, and in stage I disease. We expand our mechanistic understanding, discovering that clonal transcriptional signals are detectable before tissue invasion, act as a molecular fingerprint for lethal metastatic clones and predict chemotherapy sensitivity. Lastly, we find that ORACLE summarizes the prognostic information encoded by genetic evolutionary measures, including chromosomal instability, as a concise 23-transcript assay.

## Abstract (web summary)

Swanton and colleagues present a clonal expression signature, ORACLE, which, in combination with clinicopathological and molecular risk factors, can predict survival of patients with lung adenocarcinoma, and they prospectively validate its prognostic value.

## Main

Lung cancer is the leading cause of global cancer-related death^1^. Non-small cell lung cancer (NSCLC) accounts for 85% of cases, of which 50% are lung adenocarcinoma (LUAD)^2^. For patients with NSCLC, tumor–node–metastasis (TNM) staging is the gold standard for clinical prognostication and therapeutic decision-making. Although TNM staging is clearly associated with survival, better predictors could be found. For example, surgical resection is performed with curative intent in patients with stage I disease, yet there is a 5-year mortality rate of 15% in this population^3^. This indicates a need to address undertreatment by identifying high-risk stage I tumors that may benefit from adjuvant therapy^4^. Moreover, as computed tomography lung-cancer screening programs are adopted, the proportion of stage I diagnoses increases from around 15% to nearly 60% (ref. ^5^). Therefore, improving prognostic accuracy in early-stage LUAD is an urgent and growing clinical need.

Transcriptomic biomarkers hold the translational potential of capturing features of cancer cell aggressiveness to add a molecular dimension to prognostication. Yet, despite two decades of research, developing reliable expression biomarkers for LUAD remains a difficult task. Previously suggested biomarkers have failed to refine risk prediction beyond established clinicopathological risk factors, particularly in stage I disease^6^, and have exhibited poor reproducibility in independent validation cohorts. This was showcased by the Director’s Challenge Consortium study in which nine top research teams failed to achieve these benchmarks^7^.

**Fig. 1:**
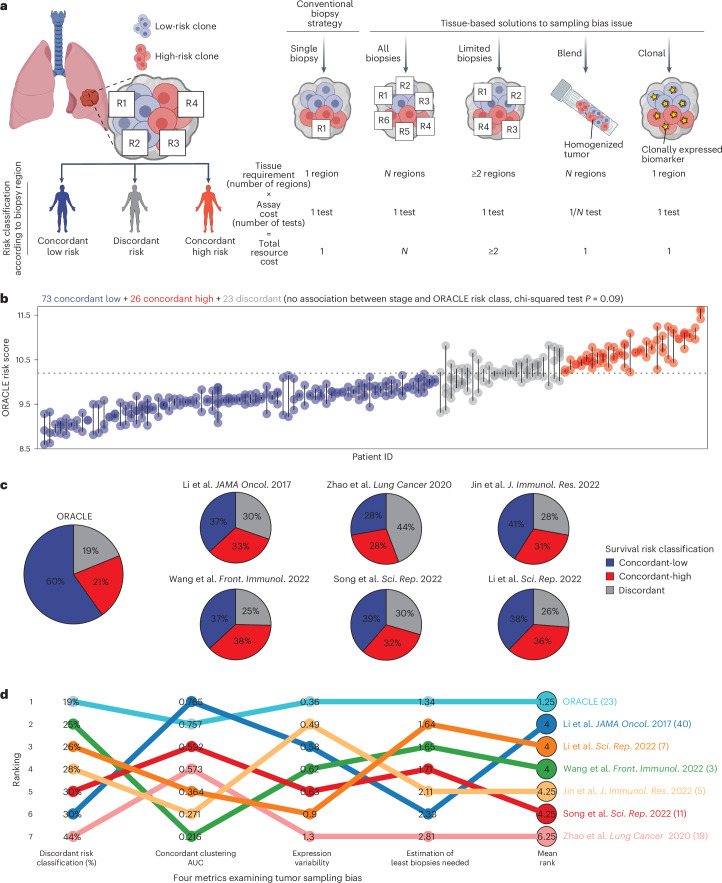
Prospective validation of tumor sampling bias. **a**, The sampling bias problem is illustrated for a lung tumor. Here, a prognostic biomarker classifies tumor regions as high risk (red) or low risk (blue). The diagnostic biopsy samples from only one tumor region (indicated by square with region number). Therefore, using the conventional strategy, the readout of molecular risk for this patient will depend entirely on where the biopsy needle is placed. Four tissue-based solutions to mitigate sampling bias are tabulated, comparing their tissue and assay requirements. Sampling and testing ‘all’ tumor regions bypasses the sampling problem, but this is the most expensive in terms of tissue and technology costs. A multibiopsy strategy, sampling a limited number of regions (four regions have been suggested for lung cancer^11^), brings down the cost while tending to capture intratumor variability. ‘Blending’ the entire tumor, and applying one test to an aliquot from the homogenized mixture, has the same cost as testing a single diagnostic biopsy but requires pathology access to the full tumor. In theory, the ‘clonal’ strategy is the most economical, providing a stable molecular readout from a single diagnostic biopsy. Created in BioRender.com. **b**, A dot plot showing the distribution of ORACLE risk scores in the TRACERx validation cohort (*n* = 122 patients with stage I–III LUAD with multiple regions available). Patients were classified into concordant low-risk (blue), concordant high-risk (red) and discordant risk (gray) groups by ORACLE. The association between ORACLE risk class and TNM stages was tested by chi-squared goodness-of-fit test in Extended Data Fig. 2b. **c**, Pie charts showing the percentages of risk groups classified by ORACLE and the other six signatures. **d**, An overview of prognostic signature ranking across four different metrics for tumor sampling bias. The mean rank of all tumor sampling bias was calculated for each signature. The name of each signature is indicated (with the number of signature genes). Source data

Previously, we quantified tumor sampling bias in the TRACERx (TRAcking non–small cell lung Cancer Evolution through therapy (Rx)) lung study (NCT01888601). We observed that pervasive intratumor heterogeneity (ITH) in lung cancer confounded prognostic signatures, with 30–40% of tumors yielding disparate prognostic scores depending upon where the biopsy needle was placed^8^. Proposed solutions to the sampling bias issue for molecular biomarkers (Fig. 1a) include: (1) bypassing sampling, by resecting the whole tumor then testing every part^9,10^; (2) sampling and pooling biopsies from different areas of a tumor to minimize artifacts from tumor heterogeneity (previous authors have suggested that four biopsies would be sufficient for lung tumors^11^ or two biopsies for glioma^12^); (3) homogenizing the entire tumor, then performing one test on the resulting mixture^13^; and (4) our previously developed strategy, identifying homogeneously (clonally) expressed markers to sample and test one biopsy per tumor^8^.

Clonal expression biomarkers may be straightforward to implement clinically, as they are compatible with existing pathology workflows and cost-effective. Accordingly, we had designed the Outcome Risk Associated Clonal Lung Expression (ORACLE) signature in TRACERx as a multiregion research cohort^8^. In retrospective validation analyses of more than 900 patients with LUAD, this biomarker maintained prognostic significance and was associated with survival independent of clinicopathological risk factors in a multivariable analysis^8^.

Here, we expand on our previous work by developing three lines of analysis related to clonal expression biomarkers in LUAD. First, we perform prospective validation of a molecular test based on cancer evolutionary principles for patients with lung cancer. Second, we expand our mechanistic understanding of clonal transcriptional signals by charting them from tumor initiation to metastasis and evaluating their association with chemosensitivity. Third, we examine the relationship between clonal RNA alterations and previously described genetic metrics of lung cancer evolution^14–17^.

## Results

### Multiregion RNA-seq data from LUAD

**Extended Data Fig. 1:**
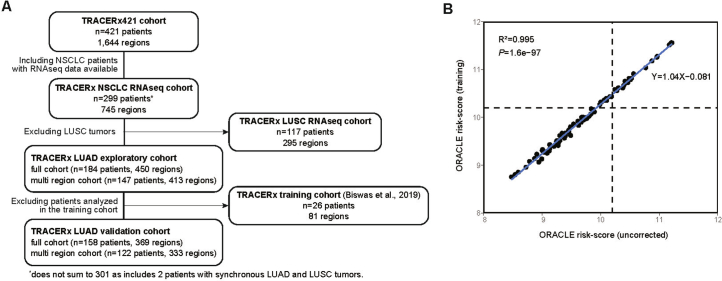
An overview of the TRACERx study. **a**, An overview of cohorts utilized in this study. A total of 421 NSCLC patients were enrolled in the TRACERx study (NCT01888601) where we focused on patients with LUAD to perform analyses on LUAD prognostic signatures. Patients involved in the training dataset published previously^8^ were removed, yielding the prospective validation cohort (n = 158). Other analyses for discovery were performed on the exploratory cohort including 184 LUAD patients. Patients with multiple regions available were included in certain analyses where specified in the text. **b**, Batch correction of ORACLE risk score using shared samples (85 regions from 27 patients) between previously published data and current data generated from an updated computational pipeline. A dot plot showing the risk scores between two data versions and risk scores were corrected using the linear regression formula. The *P* value (*P* = 1.6×10^−97^) was tested using a linear regression model and the coefficient of determination (R^2^) was shown in the graph. Source data

Previously, we utilized data from the first 100 patients recruited into the TRACERx study (TRACERx100 cohort, including 28 patients with stage I–III LUAD, 89 tumor regions) to quantify the RNA ITH of prognostic biomarkers in LUAD^8^. In this work, we leverage multiregion RNA sequencing (RNA-seq) data from an expanded cohort of patients with stage I–III LUAD recruited prospectively in the TRACERx study (Extended Data Fig. 1a). For the validation of ORACLE in an independent patient cohort, we exclude patients profiled in our previous study to yield the TRACERx validation cohort, consisting of 369 tumor regions from 158 patients. Separately, for additional exploratory analyses, we utilize the full combined set of patients, termed the TRACERx exploratory cohort, comprising 450 tumor regions from 184 patients. All primary tumor regions were sampled from treatment-naive patients. ORACLE risk scores were determined as described in the original publication^8^, applying predefined model coefficients and risk-score cutoff (Methods and Extended Data Fig. 1b).

### Benchmarking tumor sampling bias

We prospectively assessed the tumor sampling bias of ORACLE, benchmarking against comparable prognostic signatures. Tumor sampling bias was quantified using four metrics in the TRACERx validation cohort, restricting analysis to patients with multiregion RNA-seq data available (333 tumor regions from 122 patients with stage I–III LUAD; Extended Data Fig. 1a). To benchmark ORACLE, six RNA-seq-based prognostic signatures for LUAD were identified from a literature search and applied as described in their original publications (Methods and Supplementary Table 1): three signatures based on immune-related genes (Li et al.^18^, Song et al.^19^ and Jin et al.^20^), one *N*^6^-methyladenosine-related signature (Wang et al.^21^), one ER-stress signature (Li et al.^22^) and one signature derived from aberrantly expressed protein-coding genes (Zhao et al.^23^).

**Extended Data Fig. 2:**
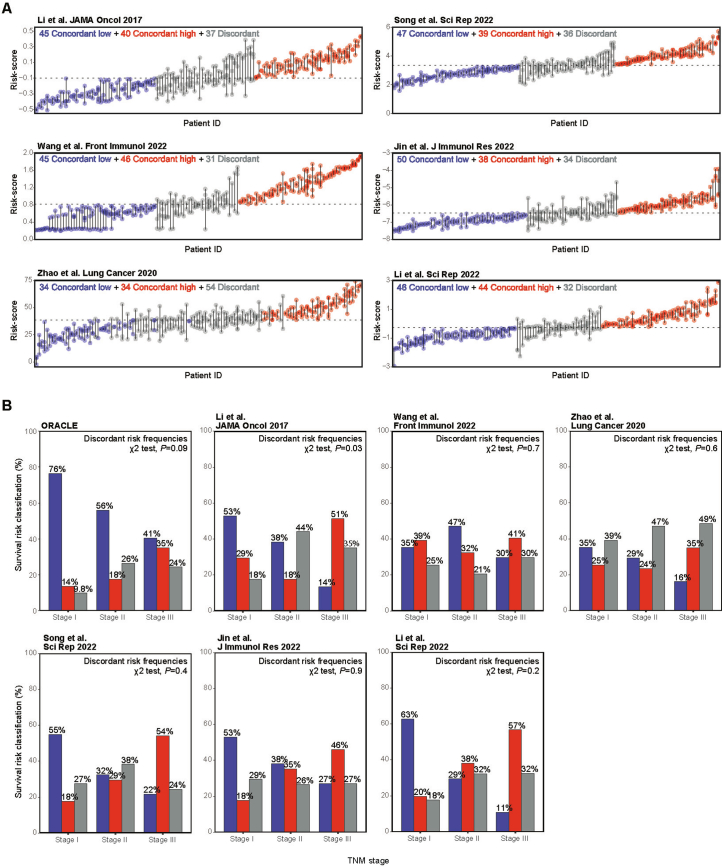
Discordance percentages of published RNA-seq prognostic signatures. **a**, Dot plots showing the distribution of risk scores for six published RNA prognostic signatures^18–23^ in the TRACERx validation cohort (n = 122 stage I-III LUAD patients with multiregion RNA-seq data available). Patients were classified into concordant low- (blue), concordant high- (red) and discordant-risk (gray) groups by each signature using median value as a cutoff. **b**, Bar plots show the percentages of risk groups classified by ORACLE risk class and the six signatures across stage I to stage III. The differences of discordant risk frequencies among tumor stages were examined using chi-squared goodness-of-fit test. Source data

First, the ORACLE signature was used to classify tumor regions as either high or low risk according to the predefined thresholds from Biswas et al.^8^. Each tumor could then be classified as concordant-low risk, concordant-high risk or discordant risk (Fig. 1a). For ORACLE, discordant risk classification was observed in 19% (23/122) of tumors compared with 25–44% across the other six signatures (Fig. 1b,c and Extended Data Fig. 2a). We also assessed whether this observation was affected by tumor stage (TNM 8th edition), finding that the discordant risk frequency for ORACLE was not significantly associated with tumor stage (chi-squared test, *P* = 0.09; Extended Data Fig. 2b).

**Extended Data Fig. 3:**
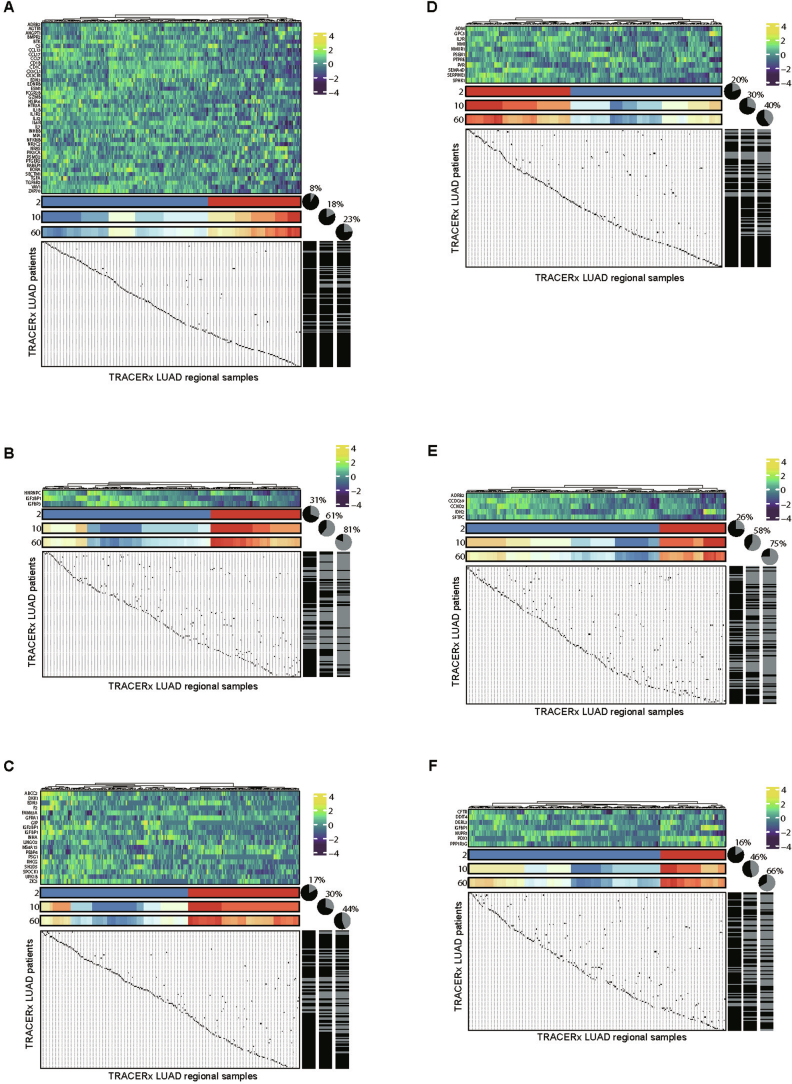
Clustering concordance of published RNA-seq prognostic signatures. A previously used hierarchical clustering method^8,24^ applied on the six published prognostic signatures is illustrated. The dendrogram and heatmap shows the clustering of tumor regions. The discordant rate (gray) was calculated as the percentage of patients with tumor regions falling into different clusters. The analysis was iterated from 1 to 122 clusters which is the maximum patient number included in this cohort. The percentage of discordant clustering was illustrated when cutting the dendrogram into 2, 10 and 60 clusters. **a**, Li et al.’s signature **b**, Wang et al.’s signature **c**, Zhao et al.’s signature **d**, Song et al.’s signature **e**, Jin et al.’s signature **f**, Li, Feng et al.’s signature. Source data

**Extended Data Fig. 4:**
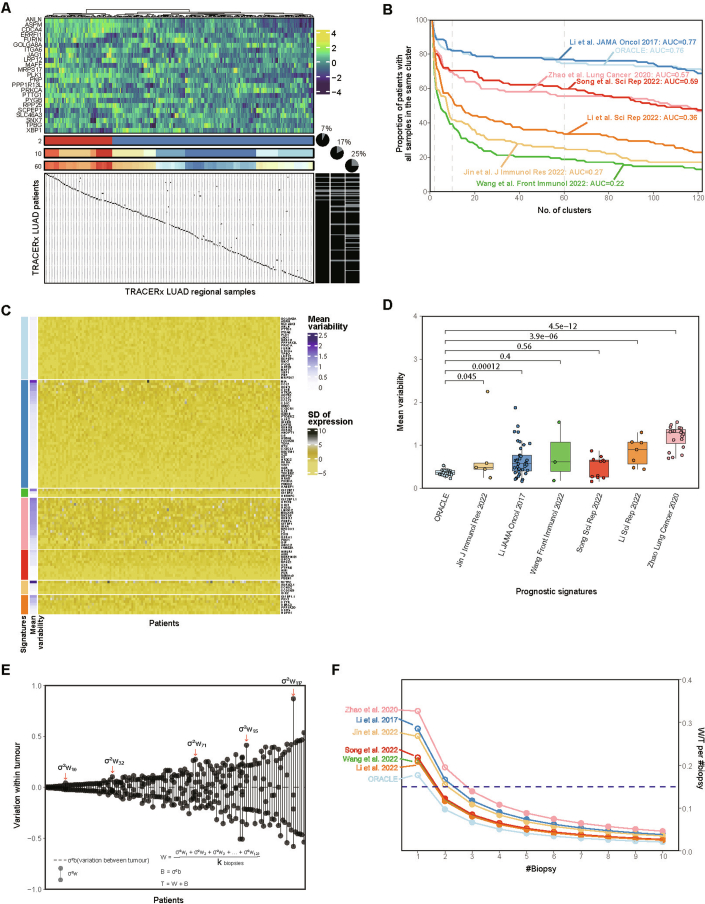
Established metrics for quantifying tumor sampling bias. **a**, The hierarchical clustering of ORACLE genes using methods described in Extended data Fig. 3. is shown. **b**, The area under the curve was calculated to represent concordant rate derived from hierarchical clustering method for ORACLE and the six published prognostic signatures. This analysis was run for 1 (100% concordant rate) to 122 clusters (the maximum number of clusters could be obtained for the cohort). Dashed line indicates the number of clusters cut in Extended data Fig. 3. **c**, A method developed by Househam et al.^25^ examining the expression variability. The heatmap shows the gene-wise standard deviation of expression across tumor regions per patient. The average of expression variability is annotated on the left. **d**, Box plot represents the distribution of mean expression variability across the signature genes for ORACLE and the six other RNA signatures in the TRACERx validation cohort (n = 122 patients with 333 tumor regions). Color for each signature is labeled as the same in panel **c**. The statistical significance was tested using a two-sided Wilcoxon rank-sum test. The center line of the boxplot indicates median and the box spans from 25th to 75th percentile. The lower and upper whiskers define the 5th and 95th percentiles, respectively. Jin et al., 2022, *P* = 0.045; Li et al., 2017, *P* = 0.00012; Wang et al., 2022, *P* = 0.4; Song et al., 2022, *P* = 0.56; Li et al., 2022, *P* = 3.9×10^−6^; Zhao et al., 2020, *P* = 4.5×10^−12^ compared with ORACLE. **e**, Estimation of minimum biopsy number needed to obtain a stable risk score using an algorithm developed by Bachtiary et al.^26^. Vertical lollipop plot represents the variance of ORACLE risk score within an individual tumor. The average value of variance within tumors divided by a certain number of biopsies (*k*) was summarized as *W*. The horizontal dashed line shows the variance between tumors involved in this cohort which is denoted as *B*. The ratio of *W* to the total variance (*T*) measures the stability of risk scores for a given signature. This method was applied to the other six signatures. **f**, Line plot represents the *W/T* per signature from one to ten biopsies. The threshold of 0.15 (horizontal dashed line) predefined in the original publication^26^ determined the intersection with the best fit line, yielding the least biopsies required to obtain a stable risk score. Source data

Second, we applied a hierarchical clustering method previously used by us and others to quantify tumor sampling bias^8,24^ (Extended Data Fig. 3). In this analysis, a larger area under the curve (AUC) value suggests more concordant classification of regions at the patient level. ORACLE exhibited an AUC value of 0.76, ranking second highest out of the seven signatures (AUC values ranging from 0.22 to 0.77; Extended Data Figs. 3 and 4a,b), with the Li et al.^18^ signature demonstrating a marginally higher AUC value (0.77).

Third, we applied a method developed by Househam et al.^25^ for capturing the intratumor expression variability of individual genes, with lower values indicating homogeneous expression (Extended Data Fig. 4c). By this metric, the genes comprising ORACLE exhibited the lowest median value at 0.36 compared with values ranging from 0.49 to 1.3 for the other signatures (Extended Data Fig. 4d), indicating greater stability in expression across tumor regions.

Lastly, motivated by the reliance on single tumor biopsies in current clinical practice, we applied a metric previously used to quantify how many biopsies would be required to obtain a stable risk-score estimate^26^ (Extended Data Fig. 4e). Using a threshold prespecified by the authors of the original study^26^, the ORACLE signature reached a stable risk-score estimate at 1.3 biopsies compared with 1.6–2.8 for the other signatures (Extended Data Fig. 4f). This suggests that ORACLE yields a more stable risk-score estimate from a single tumor biopsy.

In this prospective validation of tumor sampling bias, ORACLE achieved the best mean rank (1.25) out of seven RNA-seq-based prognostic signatures for LUAD (range 4–6.25) across four metrics for tumor sampling bias (Fig. 1d).

### Prospective validation

Next, we focused on prospective assessment of the survival association of ORACLE in the TRACERx validation cohort (*n* = 158 patients with stage I–III LUAD; Extended Data Fig. 1a).

**Fig. 2:**
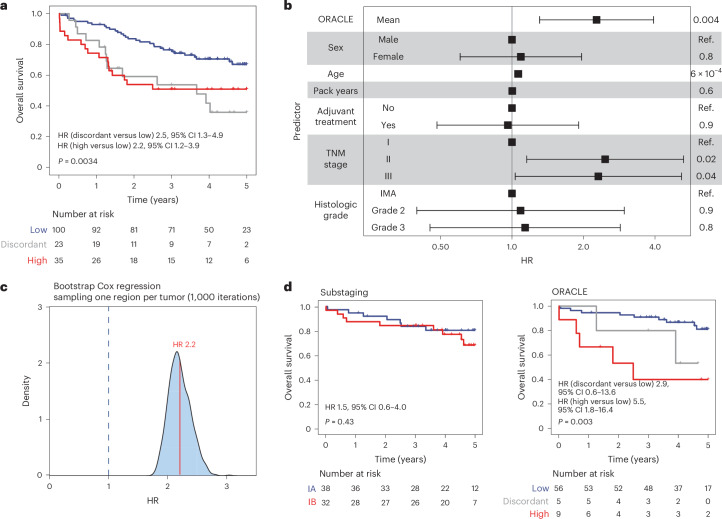
Prospective validation of survival association. **a**, A Kaplan–Meier plot showing the OS association among patients at low risk (blue), high risk (red) and discordant risk (gray) classified by ORACLE in the TRACERx validation cohort (*n* = 158 patients with stage I–III LUAD). Statistical significance was tested with a two-sided log-rank test, *P* = 0.0034. **b**, The prognostic value of ORACLE adjusted for known clinicopathological risk factors in the TRACERx validation cohort (*n* = 158 patients with stage I–III LUAD). Multivariable Cox analysis was performed incorporating the ORACLE mean risk score, patient sex, patient age, pack-years (smoking packs and duration), adjuvant treatment status, tumor stage (TNM 8th edition) and histologic grade. *P* values or baseline (Ref.) are shown for each predictor in the last column. The center box indicating HR and the error bars indicating 95% CIs are shown for each predictor on a natural log scale. IMA, invasive mucinous adenocarcinoma. **c**, The distribution of prognostic associations for ORACLE across simulation runs of a pseudo-single-biopsy cohort. One region is randomly sampled for each tumor followed by a Cox regression analysis of ORACLE risk score against OS. The density plot shows the distribution of log-scaled HR values across 1,000 simulations. **d**, The prognostic value of ORACLE for patients with stage I (TNM 8th edition) LUAD in the TRACERx validation cohort (*n* = 70). The Kaplan–Meier plots show the OS association according to clinical staging (TNM 8th edition) (*P* = 0.43) and ORACLE (*P* = 0.003). Statistical significance was tested with a two-sided log-rank test. Source data

We calculated hazard ratio (HR) values to compare ORACLE risk classes: concordant-high versus concordant-low, and discordant versus concordant-low. There was a clear association between ORACLE risk class and overall survival (OS) (Fig. 2a; concordant-high versus concordant-low HR 2.2 (95% confidence interval (CI) 1.2–3.9), discordant versus concordant-low HR 2.5 (95% CI 1.3–4.9), *P* = 0.0034).

We next examined whether the association between ORACLE and survival was independent of known clinicopathological risk factors (sex, age, smoking pack-years, adjuvant treatment status, tumor stage (TNM 8th edition) and histologic grade). Adjusted HR (HR-adj) values were calculated using a multivariable analysis in the TRACERx validation cohort (*n* = 158 patients with stage I–III LUAD; Extended Data Fig. 1a). ORACLE was used as a continuous risk measure, by calculating the mean score across regions per tumor. The ORACLE risk score was significantly associated with OS (HR-adj 2.27 (95% CI 1.3–3.9), *P* = 0.004; Fig. 2b) when adjusted for sex, age, smoking pack-years, adjuvant treatment status, tumor stage (TNM 8th edition) and histologic grade.

In clinical practice, typically only one biopsy is available per tumor to determine molecular risk scores. We generated a pseudo-single biopsy cohort to evaluate ORACLE in this context, by randomly sampling one region per tumor, calculating the risk score for that region, then testing the survival association. Running this simulation 1,000 times, the ORACLE risk score remained significantly associated with OS across every iteration (Fig. 2c, bootstrapped HR 2.2, bootstrapped CI 1.42–3.42).

**Extended Data Fig. 5:**
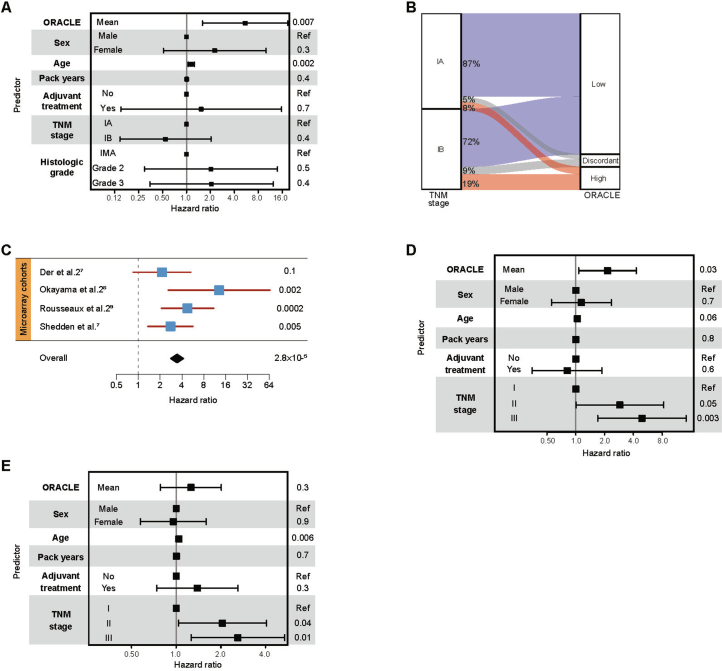
Prospective validation of survival association in stage I LUAD and using lung-cancer-specific survival and DFS. **a**, Prognostic value of ORACLE in predicting the OS in stage I subgroup (n = 70 patients with stage I LUAD) adjusted for known clinicopathological risk factors. Multivariable Cox analysis was performed incorporating the ORACLE mean risk score, patient sex, patient age, pack years (smoking packs and duration), adjuvant treatment status, tumor stage (TNM 8th edition) and histologic grade. The center box indicating hazard ratio and the error bars indicating 95% confidence intervals are shown for each predictor on a natural log scale. **b**, The percentages of stage I patients that transit from standard clinical substaging (TNM 8th edition) to ORACLE risk classification. The patients in the TRACERx validation cohort (n = 70 stage I LUAD patients) were stratified by tumor stage into stage IA (n = 38) and stage IB (n = 32) on the left and classified by ORACLE as concordant low- (n = 56), concordant high- (n = 9) and discordant risk (n = 5) groups on the right. The color shows the transition from stage I to ORACLE low- (blue), high- (red) and discordant-risk (gray) groups. **c**, Prognostic value of ORACLE in a meta-analysis across four independent cohorts of patients with LUAD (n = 580 patients with stage I LUAD). Univariate Cox analysis was performed in four microarray datasets (Shedden et al.^7^, Der et al.^27^, Okayama et al.^28^ and Rousseaux et al.^29^). The center box indicating hazard ratio and the error bars indicating 95% confidence intervals are shown for each predictor on a natural log scale. The diamond indicates the hazard ratio for the meta-analysis of the four microarray cohorts. **d**, Prognostic value of ORACLE in predicting the lung-cancer-specific death adjusted for known clinicopathological risk factors in the TRACERx validation cohort (n = 158 stage I-III LUAD patients). Multivariable Cox analysis was performed incorporating the ORACLE mean risk score, patient sex, patient age, pack years (smoking packs and duration), adjuvant treatment status and tumor stage (TNM 8th edition). The center box indicating hazard ratio and the error bars indicating 95% confidence intervals are shown for each predictor on a natural log scale. **e**, Prognostic value of ORACLE in predicting the DFS adjusted for known clinicopathological risk factors in the TRACERx validation cohort (n = 158 stage I-III LUAD patients). Multivariable Cox analysis was performed incorporating the ORACLE mean risk score, patient sex, patient age, pack years (smoking packs and duration), adjuvant treatment status and tumor stage (TNM 8th edition). The center box indicating hazard ratio and the error bars indicating 95% confidence intervals are shown for each predictor on a natural log scale. Source data

We also evaluated ORACLE specifically in patients with stage I LUAD in the TRACERx validation cohort (*n* = 70 patients with stage I LUAD), where a prognostic biomarker might have the greatest utility for adjuvant therapy use^5^. Classifying these patients according to the current clinical standard (TNM 8th edition, *n* = 38 in stage IA, *n* = 32 in stage IB), tumor substaging criteria were not prognostically informative (log-rank *P* = 0.43; Fig. 2d). By contrast, stratifying these patients into ORACLE risk classes (concordant-low *n* = 56, discordant *n* = 5, concordant-high *n* = 9) showed a significant association with OS (log-rank *P* = 0.003; Fig. 2d). The association between ORACLE risk score and OS in the stage I subgroup remained significant (HR-adj 5.48 (95% CI 1.6–18.8), *P* = 0.007; Extended Data Fig. 5a) when adjusted for sex, age, smoking pack-years, adjuvant treatment status, tumor stage (TNM 8th edition) and histologic grade. We further compared substaging classification with ORACLE risk class, finding that 8% (3/38) of patients with stage IA and 19% (6/32) of patients with stage IB were classified as ORACLE high risk (Extended Data Fig. 5b). To compare the predictive utility of ORACLE with other prognostic signatures, we calculated area under the receiver operating characteristic curve (AUROC) values, finding that the ORACLE risk score exhibited higher concordance with OS in stage I disease (AUROC 0.73) than the other six signatures (AUROC 0.59–0.72; Table 1). Lastly, a meta-analysis of four microarray datasets^7,27–29^ from other institutions revealed that ORACLE risk score was significantly associated with survival outcome in the stage I subgroup (HR 3.4 (95% CI 2.2–5.4), *P* = 2.8 × 10^−5^; Extended Data Fig. 5c), providing additional validation in external cohorts.

**Table 1:** AUROC and C index calculated for patients with stage I LUAD (*n* = 70) using survival endpoints for LUAD RNA-seq prognostic signatures

	Overall survival		Lung-cancer-specific survival		Disease-free survival	
	AUROC	C index	AUROC	C index	AUROC	C index
ORACLE	0.726	0.705	0.714	0.741	0.588	0.587
Li et al. *JAMA Oncol.* 2017	0.715	0.717	0.553	0.603	0.661	0.66
Song et al. *Sci. Rep*. 2022	0.705	0.664	0.692	0.685	0.615	0.604
Zhao et al. *Lung Cancer* 2020	0.674	0.598	0.576	0.597	0.629	0.62
Li et al. *Sci. Rep.* 2022	0.615	0.595	0.635	0.654	0.546	0.529
Wang et al. *Front. Immunol.* 2022	0.611	0.598	0.642	0.626	0.607	0.592
Jin et al. *J. Immunol. Res.* 2022	0.593	0.556	0.558	0.528	0.576	0.567

### ORACLE as a biomarker of invasive and metastatic potential

Previously we had observed that ORACLE risk scores were significantly higher in metastatic samples from patients with LUAD, suggesting that ORACLE may serve as a signature for metastatic potential^8^. We wished to extend this finding by investigating whether high-risk clonal expression changes are present before tissue invasion and whether the lethal disseminating clone is detectable in the transcriptome of the primary tumor.

**Fig. 3:**
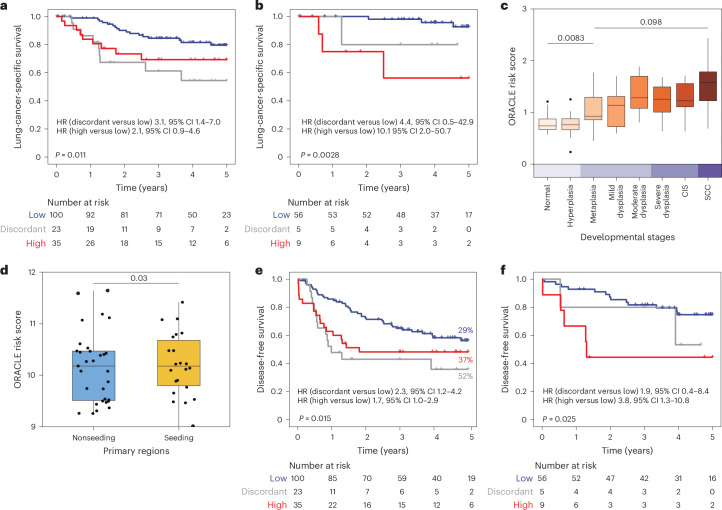
ORACLE as a marker of invasive and metastatic potential. **a**,**b**, Kaplan–Meier plots showing the lung-cancer-specific survival association among patients at low risk (blue), high risk (red) and discordant risk (gray) classified by ORACLE in the TRACERx validation cohort (*n* = 158 patients with stage I–III LUAD, *P* = 0.011) (**a**) and in stage I subgroup (*n* = 70 patients with stage I LUAD, *P* = 0.0028) (**b**). Statistical significance was tested with a two-sided log-rank test. **c**, ORACLE risk scores in 8 histological stages in a published dataset of preinvasive lung lesions (122 biopsies from 77 patients). Each histological stage was further grouped into different lesion grades according to the original article (Methods). The statistical significance was assessed by a linear mixed-effects model setting histological stages as fixed effect and accounting for individual patients as a random effect. No correction was made for multiple comparisons among developmental stages. Metaplasia versus normal stage, *P* = 0.0083; SCC versus metaplasia, *P* = 0.098. **d**, ORACLE risk scores compared between primary regions seeding and nonseeding metastatic clones determined by the phylogenies in the TRACERx exploratory cohort (*n* = 17 tumors including 22 seeding regions and 31 nonseeding regions). The statistical significance was tested with a linear mixed-effects model using primary tumor regions as a fixed effect and accounting for individual patients as a random effect, *P* = 0.03. **e**, A Kaplan–Meier curve showing the DFS of ORACLE in the TRACERx validation cohort (*n* = 158 patients, with 54 of them having relapse). The percentages of patients developing relapse in each ORACLE risk class are annotated. Statistical significance was tested with a two-sided log-rank test. **f**, A Kaplan–Meier curve showing the DFS of ORACLE in stage I subgroup (*n* = 70 patients with stage I LUAD). The statistical significance was tested by a two-sided log-rank test. For **c** and **d**, the center line of the boxplot indicates the median and the box spans from the 25th to 75th percentile. The lower and upper whiskers define the 5th and 95th percentiles, respectively. Source data

First, we tested whether ORACLE, as a lung cancer marker, predicted lung-cancer-specific survival in the TRACERx validation cohort (*n* = 158 patients with stage I–III LUAD). A significant association was found between ORACLE risk class and lung-cancer-specific survival (concordant-high versus concordant-low HR 2.1 (95% CI 0.9–4.6), discordant versus concordant-low HR 3.1 (95% CI 1.4–7.0), *P* = 0.011; Fig. 3a). The association between ORACLE risk score and lung-cancer-specific survival remained significant in a subgroup analysis of patients with stage I disease (log-rank *P* = 0.0028; Fig. 3b) and when controlling for clinicopathological risk factors (HR-adj 2.15 (95% CI 1.1–4.3), *P* = 0.03; Extended Data Fig. 5d). ORACLE risk score was also a better predictor of lung-cancer-specific survival in stage I LUAD (AUROC 0.71) compared with the other six prognostic signatures (AUROC 0.55–0.69; Table 1).

Next, to track the transition from normal tissue to cancer, we examined ORACLE risk scores across eight histological stages (*n* = 77 patients, including 27 normal tissues, 15 hyperplasia, 15 metaplasia, 13 mild dysplasia, 13 moderate dysplasia, 12 severe dysplasia, 13 carcinoma in situ (CIS) and 14 squamous cell carcinoma (SCC))^30^. Charting ORACLE risk scores by developmental stages revealed an increase in expression from normal to metaplasia (linear mixed-effects model *P* = 0.0083; Fig. 3c).

We evaluated whether a lethal disseminating phenotype could be detected in the transcriptome of primary tumor regions harboring a metastatic subclone. Leveraging paired primary-metastasis phylogenies^31^ within the TRACERx exploratory cohort, we superimposed ORACLE risk scores onto metastatic competence at the level of tumor regions (53 tumor regions from *n* = 17 patients with stage I–III LUAD with paired metastasis-seeding regions (22) and non-metastasis-seeding regions (31)). In this analysis, seeding regions displayed significantly higher ORACLE risk scores than nonseeding regions (linear mixed-effects model *P* = 0.03; Fig. 3d). To examine whether ORACLE risk was informative for predicting early systemic dissemination, we assessed the time to relapse or death using disease-free survival (DFS) in the TRACERx validation cohort (*n* = 158 patients with stage I–III LUAD). A significant association was found between ORACLE risk class and DFS (concordant-high versus concordant-low HR 2.3 (95% CI 1.2–4.2), discordant versus concordant-low HR 1.7 (95% CI 1.0–2.9), *P* = 0.015; Fig. 3e). We also performed a subgroup analysis finding that ORACLE risk class was significantly associated with DFS in patients with stage I disease (*P* = 0.025, Fig. 3f; ORACLE AUROC 0.59, other signatures AUROC values 0.55–0.66; Table 1). The association between ORACLE risk score and DFS was not significant when adjusted for clinicopathological risk factors (HR-adj 1.3 (95% CI 0.8–2.0), *P* = 0.3; Extended Data Fig. 5e). Relapse rates at 5 year follow-up were higher for concordant-high (37%, 13/35) and discordant (52%, 12/23) risk classes than for the concordant-low (29%, 29/100) group (Fig. 3e). Notably, the rate of progression was more rapid in the high-risk (median DFS 1.8 years) and discordant-risk groups (median DFS 0.99 years) compared with the low-risk group (median DFS not reached).

Overall, these data indicate that high-risk clonal expression changes are present in preinvasive lesions, remain detectable in primary tumors that achieve early systemic dissemination and can serve as a molecular fingerprint for the lethal metastasizing subclone.

### ORACLE delineates chemosensitive cells

Predicting patient benefit from adjuvant chemotherapy is a major challenge in early-stage NSCLC^32,33^. We therefore investigated the utility of ORACLE for identifying chemosensitivity in treatment-naive patients.

**Fig. 4:**
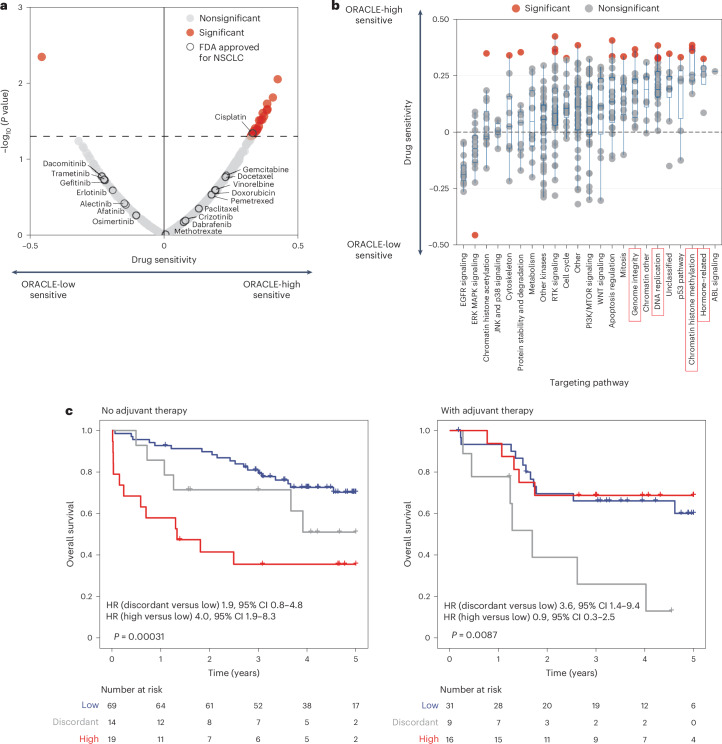
ORACLE delineates chemosensitive cells. **a**, A volcano plot showing the correlation between ORACLE risk scores and the sensitivity to anticancer drugs available from the GDSC database (*n* = 37 LUAD cell lines; 359 compounds; Methods). The analysis was performed using Spearman correlation with the coefficient (*ρ*) labeled on the *x* axis and the *P* value labeled on the *y* axis. Drugs labeled in red indicate a significant association with ORACLE risk scores. FDA-approved drugs for NSCLC are annotated and circled with black color. **b**, A dot plot showing the distribution of Spearman coefficients for drugs categorized according to their targeting pathways. The targeting pathways for each drug (359 compounds) were obtained from the GDSC database^34^. Drugs showing significant association with ORACLE risk scores are labeled in red. The center line of the boxplot indicates the median, and the box spans from the 25th to 75th percentile. The lower and upper whiskers define the 5th and 95th percentiles, respectively. **c**, Kaplan–Meier curves of ORACLE as a predictive marker for response to adjuvant therapies, dividing patients by the adjuvant treatment status in the TRACERx validation cohort (*n* = 102 without adjuvant therapy, *n* = 56 with adjuvant therapy). The statistical significance was tested with a two-sided log-rank test, no adjuvant therapy *P* = 0.00031 and with adjuvant therapy *P* = 0.0087. Source data

**Extended Data Fig. 6:**
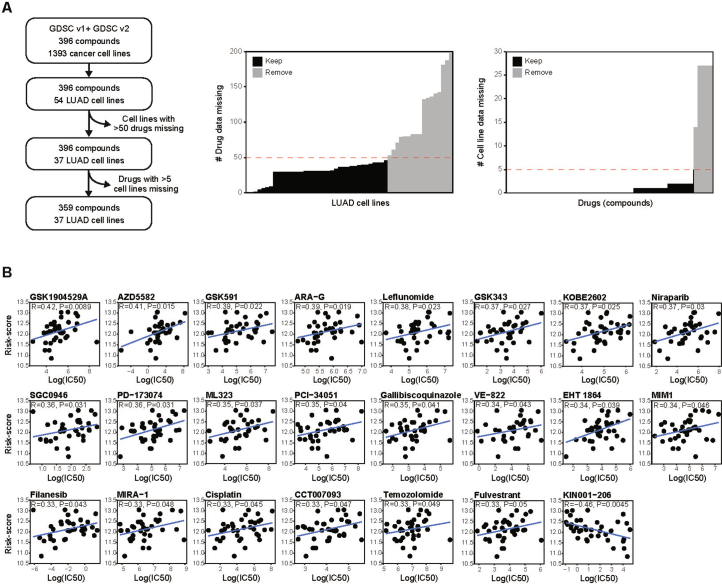
Anticancer drug screening in vitro. **a**, Flow diagram represents the steps for filtering cell lines and compounds obtained from GDSC and CCLE database^34,35^ with missing data (n = 54 LUAD cell lines; 396 compounds). Cell lines with more than 50 compound data missing were first removed, yielding 37 cell lines. Compounds with more than 5 cell line data missing were then removed, yielding 359 compounds. **b**, The association of ORACLE risk score and anticancer drug response determined by half-maximal inhibitory concentration (IC_50_). Drugs with significant association (see Fig. 4a) are shown in this figure. Spearman correlation coefficients and *P* values are shown for each compound. Source data

First, we examined the relationship between ORACLE risk score and sensitivity to cytotoxic or targeted chemotherapies by leveraging drug sensitivity screening data in the Genomics of Drug Sensitivity in Cancer (GDSC) database^34^, which are linked to transcriptomic profiles for LUAD cell lines in the Cancer Cell Line Encyclopedia^35^. Cell lines and compounds with missing data were filtered (Methods and Extended Data Fig. 6a). For each compound, we ranked LUAD cell lines according to ORACLE risk score, then examined the correlation with drug response determined by half-maximal inhibitory concentration (IC_50_) (Extended Data Fig. 6b); multiple-testing correction was not applied for this exploratory analysis. Focusing on the 17 the US Food and Drug Administration (FDA)-approved drugs for NSCLC, only cisplatin was significantly correlated with efficacy in ORACLE high-risk cell lines (Fig. 4a, *P* = 0.045, Spearman coefficient 0.33). Furthermore, across all compounds screened, responses to 23 drugs positively correlated with ORACLE risk score. GSK1904529A, a small molecule inhibiting insulin-like growth factor-1 receptor (IGF-1R) harbored the strongest association with ORACLE risk score (*P* = 0.0089, Spearman coefficient 0.42). Notably, the main mechanism of GSK1904529A is cell cycle arrest^36^ and we have previously observed cell cycle genes to be enriched among clonal transcriptional signals^8^. Only one drug, a B-Raf serine-threonine kinase (BRAF) inhibitor KIN001-206, was negatively correlated with ORACLE risk score (*P* = 0.0045, Spearman coefficient −0.46; Fig. 4a and Extended Data Fig. 6b). By categorizing therapeutic compounds on the basis of targeted pathways, we identified four pathways—hormone-related, chromatin histone methylation, DNA replication and genome integrity—where all compounds exhibited positive correlation with ORACLE risk. By contrast, compounds involved in inhibition of epidermal growth factor receptor (EGFR) signaling tended to display a negative correlation with ORACLE risk (Fig. 4b).

**Extended Data Fig. 7:**
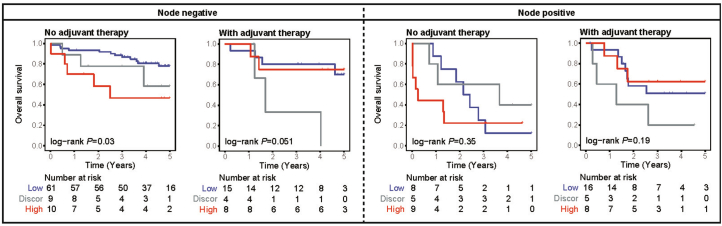
Prediction of adjuvant therapy response. ORACLE as a predictive marker of response to adjuvant therapies stratified by nodal status in the TRACERx validation cohort (*n* = 158 patients with stage I-III LUAD). Statistical significance was tested using a two-sided log-rank test. Node negative no adjuvant therapy, *P* = 0.03; node negative with adjuvant therapy, *P* = 0.051; node positive no adjuvant therapy, *P* = 0.35; node positive with adjuvant therapy, *P* = 0.19. Source data

To test whether adjuvant chemotherapy modulates the prognostic information captured by ORACLE, we divided patients from the TRACERx validation cohort into two subgroups according to their adjuvant treatment status (*n* = 102 non-adjuvant-treated, *n* = 56 adjuvant-treated; patients with stage I–III LUAD) and then stratified by ORACLE risk class (Fig. 4c). In the non-adjuvant-treated subgroup, a significant difference in OS rates was observed between ORACLE concordant-high risk patients (5-year OS rate 36%) and concordant-low risk patients (5-year OS rate 70%) (Cox regression *P* = 0.0001, HR 4.0 (95% CI 1.9–8.3)). By contrast, in the adjuvant-treated subgroup, there was no difference in OS rates between ORACLE concordant-high risk patients (5-year OS rate 69%) and concordant-low risk patients (5-year OS rate 60%) (Cox regression *P* = 0.8, HR 0.9 (95% CI 0.3–2.5)). This result, wherein ORACLE high-risk classification was more discriminatory among patients who did not receive adjuvant therapy, remained consistent when controlling for nodal status in this cohort of patients (Extended Data Fig. 7).

Taken together, these in vitro drug screen data and exploratory clinical data suggest that ORACLE high-risk LUAD tumors may be sensitive to platinum chemotherapy agents.

### ORACLE as a summary metric of lung cancer evolution

**Fig. 5:**
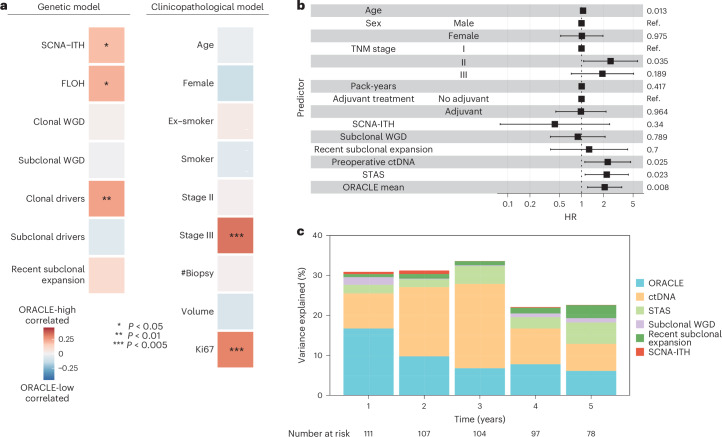
ORACLE as a summary metric of lung cancer evolution. **a**, Clinicopathological and genetic correlates with ORACLE magnitude in the TRACERx exploratory cohort (*n* = 184 patients with stage I–III LUAD). A multiple linear model was applied separately for clinicopathological or genetic features (Methods). #Biopsy, number of biopsies. Each predictor is shown in the column with its model coefficient represented by color scales and labeled with significance (*P < 0.05, **P < 0.01, ***P < 0.005). For categorical variables including female, ex-smoker and smoker, stage II and stage III, the references are male, non-smoker and stage I, respectively. No correction was made for multiple comparisons. **b**, The OS association of six biomarkers identified in the TRACERx study^14^ was examined in the TRACERx exploratory cohort (*n* = 111 patients with stage I–III LUAD with all biomarker data available). Multivariable Cox analysis was performed on ORACLE, recent subclonal expansion, SCNA-ITH, subclonal WGD, detection of preoperative ctDNA status and STAS, adjusted for known clinicopathological risk factors. *P* values or baseline (Ref.) are shown for each predictor in the last column. The center box indicating HR and the error bars indicating 95% CIs are shown for each predictor on a natural log scale. **c**, The percentages of variation of survival outcome explained by the six TRACERx biomarkers were examined by a generalized linear model. Source data

To explore the underpinnings of clonal expression signals, we evaluated clinicopathological correlates in the TRACERx exploratory cohort (*n* = 184 patients with stage I–III LUAD, Extended Data Fig. 1a; Methods). The mean ORACLE risk score was calculated as a summary measure per tumor, for use in multiple linear regression analyses. We identified two clinicopathological features that were significantly associated with ORACLE risk scores: tumor stage III (*P* = 0.002), as shown previously^8^, and Ki67 (*P* = 0.0009; Fig. 5a).

**Extended Data Fig. 8:**
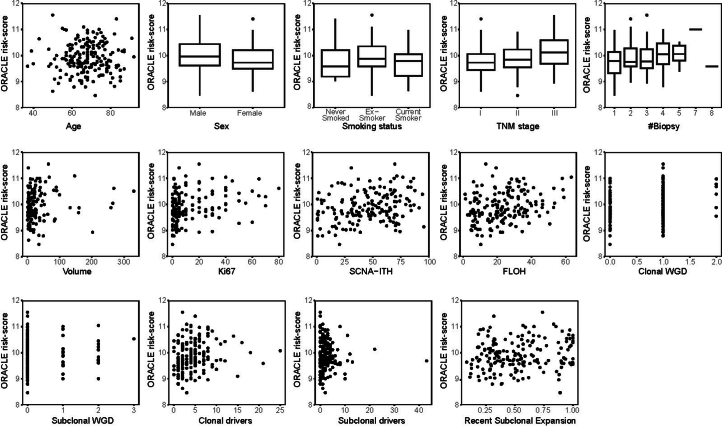
The association of ORACLE with genetic evolutionary metrics. Scatter plots and boxplots show the mean of ORACLE risk score summarized per tumor in the TRACERx exploratory cohort (n = 184 patients with stage I-III LUAD) and the correlation with seven clinicopathological and seven genetic features. The center line of the boxplot indicates median and the box spans from 25th to 75th percentile. The lower and upper whiskers define the 5th and 95th percentiles, respectively. Source data

We next examined genetic features defined in the TRACERx study^14^: whole-genome doubling (WGD) events, chromosomal complexity (fraction of loss of heterozygosity, FLOH), somatic copy-number alteration (SCNA)-ITH, and clonal and subclonal mutations in driver genes. The mean ORACLE risk score per tumor significantly correlated with SCNA-ITH (*P* = 0.02), FLOH (*P* = 0.01) and the number of clonal driver mutations (*P* = 0.009; Fig. 5a and Extended Data Fig. 8).

**Extended Data Fig. 9:**
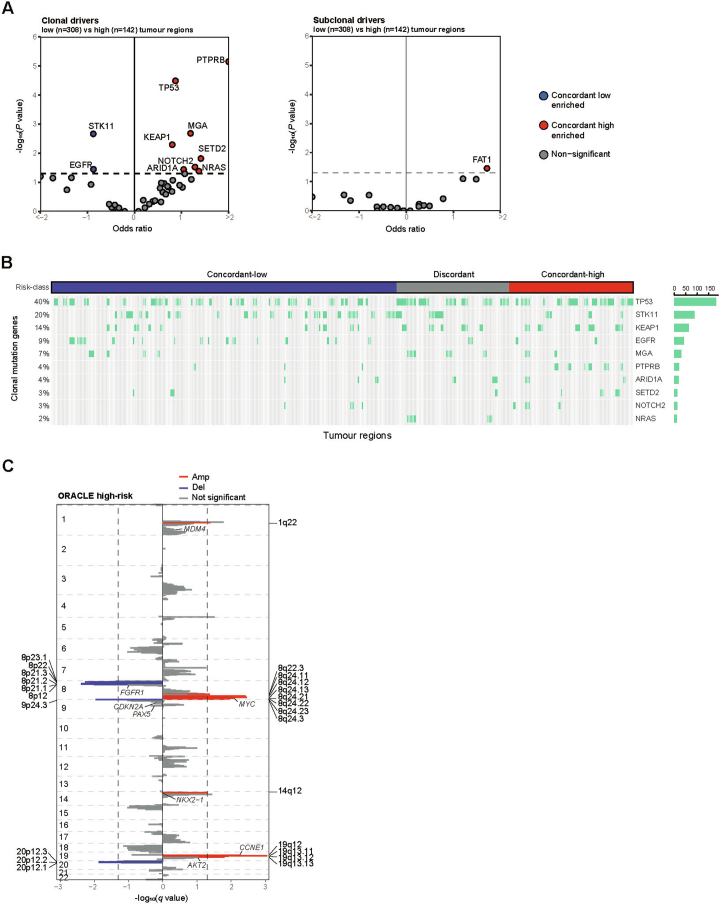
Somatic mutations and copy number alterations underlying clonal expression magnitude. **a**, Frequencies of clonal (left) and subclonal (right) driver mutations at gene level compared between high- and low-risk tumor regions in the TRACERx exploratory cohort (n = 142 high-risk and n = 308 low-risk tumor regions from 184 patients with stage I-III LUAD). The scatter plot shows the odds ratio obtained by a two-sided Fisher’s exact test for each gene mutation. A *P* value of 0.05 was indicated by the horizontal dashed line. **b**, Oncoprint shows the frequencies of clonal mutations in 10 driver genes that were enriched in ORACLE low-risk and high-risk groups. The column represents the regions across patient tumors in the TRACERx exploratory cohort (n = 184 patients with stage I-III LUAD with 450 region samples). **c**, The genome-wide SCNAs identified using GISTIC2.0 (Methods). For a given genome region, the G-score difference was calculated between ORACLE low-risk and high-risk cohorts to identify loci with positive selection. The plot shows the false-discovery rate (*q* value) of the G score in the high-risk cohort. Chromosome segments with significant positive selection (G-score difference >0 and *q* value < 0.05) are shown in red for amplification and blue for deletion. Vertical dashed lines indicate the threshold of a false-discovery rate (*q* value) equal to 0.05. The driver SCNAs, as listed in our previous study^14^, located in the chromosome arm harboring detected cytobands are highlighted. Source data

To contextualize ORACLE-associated somatic alterations to specific driver genes, we compared frequencies of each driver at gene level between low-risk (*n* = 308) and high-risk (*n* = 142) tumor regions in the TRACERx exploratory cohort (*n* = 184 patients with stage I–III LUAD). ORACLE high-risk tumor regions were enriched (*P* < 0.05, odds ratio (OR) >1) in clonal mutations occurring in eight driver genes (*PTPRB*, *TP53*, *MGA*, *KEAP1*, *SETD2*, *NOTCH2*, *ARID1A* and *NRAS*) and depleted (*P* < 0.05, OR <1) in tumor regions with clonal mutations of *EGFR* or *STK11* genes (Extended Data Fig. 9a,b). Performing the same analysis for subclonal SNVs in driver genes revealed *FAT1* gene enrichment in ORACLE high-risk regions (*P* = 0.03, OR 5.6), possibly due to this gene’s putative role in maintaining genome integrity^37^.

As ORACLE risk score reflected chromosomal instability and complexity, we wished to identify recurrent SCNA events using GISTIC2.0^38^ to compare positive-selection scores (G score) between ORACLE concordant high-risk and low-risk patients in the TRACERx exploratory cohort (*n* = 158 patients with stage I–III LUAD with concordant high- or low-risk classification, Extended Data Fig. 1a; Methods). Identifying cytobands associated with ORACLE high-risk (G-score difference >0, false discovery rate *q* < 0.05), significant enrichment was observed for 14 amplifications (Extended Data Fig. 9c): 1q22, 8q22.3, 8q24.11-13, 8q24.21-23, 8q24.3, 14q12, 19q12 and 19q13.11-13. These amplified chromosome arms include the *NKX2-1* gene (which encodes thyroid transcription factor 1 (TTF1) an established histopathology marker for LUAD) as well as *MDM4*, *MYC*, *CCNE1* and *AKT2*. Significant enrichment was also observed for ten cytoband deletions (8p23.1, 8p22, 8p21.3-1, 8p12, 9p24.3 and 20p12.3-1), including *FGFR1*, *CDKN2A* and *PAX5* genes (Extended Data Fig. 9c).

Six biomarkers have been identified as associated with survival in the TRACERx study: recent subclonal expansion^14^, subclonal WGD^14^, preoperative circulating tumor DNA (ctDNA)^15^, SCNA-ITH^16^, spread through airway spaces (STAS)^17^, and ORACLE^8^. We performed multivariable analysis to quantify the comparative prognostic information between these biomarkers, including clinical risk factors in the TRACERx exploratory cohort (*n* = 111 patients with stage I–III LUAD with all biomarker data available). Three biomarkers remained significantly associated with OS (Fig. 5b): ORACLE (*P* = 0.008, HR 2.06), STAS (*P* = 0.023, HR 2.2) and preoperative ctDNA (*P* = 0.025, HR 2.27). We also calculated the percentage variance explained (PVE) encoded by each of these six biomarkers to examine the dynamics of their prognostic association (Fig. 5c). This analysis showed that ORACLE risk score was responsible for the greatest variance in OS outcomes in the first year after LUAD diagnosis (PVE 16.7%) and remained informative (PVE range 6.1–9.7%) alongside ctDNA and STAS over a 5-year follow-up period.

Overall, these results suggest that clonal expression signals correspond to single-nucleotide variants (SNVs) and SCNAs occurring early in tumor evolution. Further, genetic evolutionary metrics previously identified in the TRACERx study (SCNA-ITH, FLOH and clonal drivers) were captured by ORACLE as a simple 23-transcript assay. Lastly, ORACLE, preoperative ctDNA and STAS encoded complementary forms of prognostic information.

## Discussion

Tissue biopsy is the gold standard for cancer diagnosis. The typical single-site needle biopsy samples less than 1% of the primary tumor mass^13^, failing to capture the full extent of genetic and transcriptomic ITH within individual tumors^14,39^. To address this sampling bias problem, we previously reported the development of a clonal expression biomarker (ORACLE), which is associated with OS outcomes in retrospective cohorts^8^.

Here, we prospectively evaluated ORACLE, recognizing cancer as an evolutionary disease to refine molecular prognostication in patients with NSCLC. In a comparison against existing LUAD RNA-seq prognostic signatures, ORACLE was prospectively validated as the top-ranked signature across four metrics for tumor sampling bias. Importantly, the association between ORACLE and OS was prospectively validated, remaining significant in multivariable analysis with known clinicopathological risk factors and in a subgroup analysis of stage I disease.

We wished to gain a deeper understanding of the clinical utility of ORACLE. Simulation of a pseudo-single biopsy cohort suggested that ORACLE remains informative in the clinical setting where tissue samples for molecular tests are usually limited^40^. The association between ORACLE and clinical outcomes was significant for lung-cancer-specific survival and DFS. As an RNA marker, ORACLE complemented the use of liquid biopsy (ctDNA) and pathology (STAS) markers to predict 5-year survival outcomes.

Lastly, we uncovered mechanism-based insights into ORACLE. Clonal transcriptional signals were ‘hard-wired’ through the acquisition of SNVs and SCNAs occurring early in tumor evolution and also delineated metastatic seeding from nonseeding primary tumor regions. These data may suggest that clonal expression biomarkers might be further developed to stratify preinvasive lesions for early intervention before systemic dissemination^41,42^. ORACLE also correlated with genetic measures of chromosomal instability and complexity. This may explain the observed relationship between ORACLE and sensitivity to chemotherapy agents (in particular, cisplatin), as chromosomally unstable tumors are hypothesized to be prone to genomic catastrophe and, hence, optimal for cytotoxic therapy^43^. Indeed, recent data support the utility of chromosomal instability signatures for predicting chemotherapy treatment response^44^.

**Extended Data Fig. 10:**
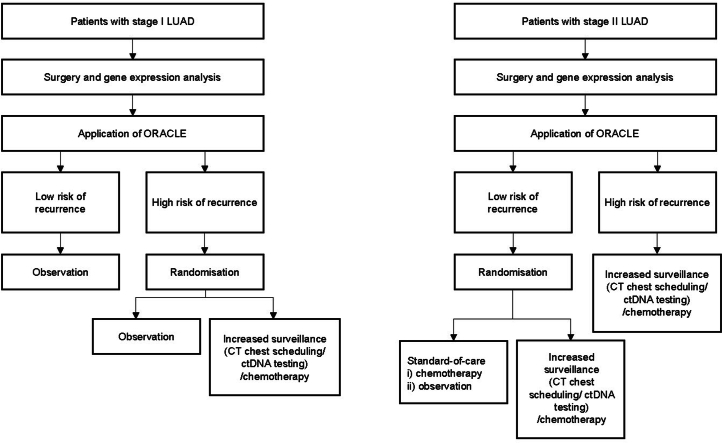
Future applicability of ORACLE in clinical practice. The possible design of prospective clinical trials to evaluate the performance of ORACLE to guide the adjuvant chemotherapy in high-risk stage I patients and monitor the outcome in low-risk stage II patients. LUAD = lung adenocarcinoma.

Future work in larger cohorts will test if ORACLE can integrate with substaging criteria to refine risk stratification within stage I disease and to validate a link between ORACLE and chemosensitivity. Breast cancer trials have prospectively evaluated the use of RNA markers to refine risk stratification for chemotherapy, thereby reducing overtreatment^45,46^. A similar approach, designing a randomized phase III trial comparing observation versus chemotherapy or closer surveillance for ORACLE high-risk tumors, may similarly move the needle for precision diagnostics in lung cancer (Extended Data Fig. 10). Moreover, the future development of a clinical-grade RNA assay^45–47^ may bypass the limitations of RNA-seq as a research-grade technology to enable real-time clinical implementation^48^.

Future work might also extend the utility of clonal expression biomarkers beyond prognostication in LUAD. We note that the method reported in our original study to derive clonally expressed genes^8^ has successfully transferred to other cancer types^49–53^. In addition, multiregion analyses suggest that existing expression-based predictive biomarkers for checkpoint immunotherapy are subject to tumor sampling bias^54^. This may suggest that deriving a clonal expression biomarker capturing the immuno-oncological status of a patient with NSCLC could help refine prediction of immune checkpoint blockade efficacy^55^.

ORACLE has been designed as a pragmatic solution to the sampling bias problem, applied to ‘bulk’ RNA extracted from single-site needle samples in the clinical setting. It has been suggested that, for a subset of tumors, prognosis is inherently difficult to predict due to low-penetrant subclones that are undetectable in bulk profiling^56^. For accurate diagnostic classification in these cases, identifying the lethal subclone may require multiregion^57–59^ or single-cell^60^ sampling strategies.

## Methods

### TRACERx cohort, sample collection and sequencing

The TRACERx study (NCT01888601) is a prospective observational cohort study aiming to transform our understanding of NSCLC; it has been approved by an independent research ethics committee (NRES Committee London) (13/LO/1546). Written informed consent was mandatory and obtained from all participants. The cohort used in this study consists of the first 421 patients who had multiple regions sampled from the same tumor to obtain DNA and RNA profiles for subsequent analyses. Sex and gender were not considered in the study design, the cohort comprised 233 (55%) men and 188 (45%) women, and all available individuals were included in each analysis. The TRACERx421 cohort (1,644 tumor regions from *n* = 421 patients), as previously reported^14^, was accessed for this study, with cohort selection as follows (Extended Data Fig. 1a). Including patients with NSCLC with RNA-seq data available yielded the TRACERx NSCLC RNA-seq cohort (745 tumor regions from *n* = 299 patients). Excluding LUSC tumors (295 regions from *n* = 117 patients) and synchronous primary tumors (*n* = 4 patients, ‘tumor 1’ IDs were included and ‘tumor 2’ IDs were excluded^14^) yielded the TRACERx LUAD exploratory cohort (450 tumor regions from *n* = 184 patients). To obtain an independent validation cohort, patients that were analyzed in the previous training cohort^8^ (81 tumor regions from *n* = 26 patients with stage I–III LUAD; the number diverges from the original study (*n* = 28 patients, 89 regions)^8^ due to sample dropout with updated TRACERx421 pipeline and cohort criteria) were excluded, yielding the TRACERx LUAD validation cohort (369 tumor regions from *n* = 158 patients). DNA and RNA was extracted using AllPrep DNA/RNA Mini Kit (Qiagen). Extracted DNA and RNA was assessed for integrity by TapeStation (Agilent Technologies). Whole-exome sequencing was performed on Illumina HiSeq 4000 or HiSeq 2500 platforms. Whole-RNA (RiboZero-depleted) paired-end sequencing was performed using an Illumina HiSeq 4000 platform. RSEM package (version 1.3.3) was used to quantify transcript counts and transcript per million (TPM) values^14,17,31,39^. Genes with expression value less than 1 TPM in at least 20% of samples were filtered out. The counts were normalized by variance-stabilizing transformation by the DESeq2 package (version 1.42.0)^61^.

### Calculating ORACLE risk scores

ORACLE risk scores were calculated as described in the original publication^8^. For each sample, each of the 23 signature genes was weighted by the model coefficient developed in the training cohort, then these values were summed to derive a risk score. ORACLE risk scores were then dichotomized using a previously defined risk-score threshold (10.199) to classify samples into low- or high-risk groups. The model coefficients are specified in Supplementary Table 5 of the original publication^8^.

### Batch correction for RNA-seq preprocessing pipeline versions

The computational pipeline for generating TRACERx RNA-seq data has been updated to the Nextflow pipeline^39^ compared with the original pipeline used in the previous study^8^. Therefore, the count values of the same samples generated by the two pipelines are technically different. To ensure the same baseline and compatibility of a predefined ORACLE risk-score cutoff with the current cohort, we performed a batch correction. A linear regression model was fit between the ORACLE risk score of shared samples generated from the original and current pipelines (85 tumor regions in 27 patients). This yielded a conversion formula, and the ORACLE risk score was corrected as shown below (Extended Data Fig. 1b).

$${{\mathrm{Corrected}}\; {\mathrm{risk}}\; {\mathrm{scores}}}={{\mathrm{risk}}\; {\mathrm{scores}}}\times 1.04-0.081$$

### Identification of LUAD RNA-seq prognostic signatures

Two RNA-seq prognostic signatures were identified in the previous study^8^. Of those, the TPM-based signature, Li et al.^18^, was selected for the analysis. Here, we used the same method as in the previous study to further identify five RNA-seq signatures^18–23^. In brief, articles describing RNA-seq prognostic signatures for LUAD were identified by literature searching on PubMed and were manually reviewed. Only signatures with a full list of genes and model coefficients specified in the articles were included for subsequent analyses.

### Tumor sampling bias metrics

Four metrics were used to measure tumor sampling bias across RNA-seq prognostic signatures:

1. The discordant rate was calculated as the percentage of patients who had regions classified as both high risk and low risk within a tumor.
2. The clustering concordance was calculated as described by Gyanchandani et al.^24^. Tumor regions were clustered on the basis of the gene expression of a given prognostic signature using Manhattan distance and the Ward.D2 method. The concordant rate was quantified by the percentage of patients with all regions falling in the same cluster. This analysis was iterated from 1 to 122 clusters (the maximum number of clusters was set as the total number of patients in the multiregion TRACERx validation cohort).
3. For a given signature gene, the expression variability was quantified as the standard deviation of expression among tumor regions from each patient. The mean variability per signature was calculated as the average expression variability across patients in the TRACERx validation cohort.
4. Bachtiary et al.^26^ previously developed a method to quantify total expression heterogeneity. In brief, the expression variance (*σ*^2^) within an individual tumor (*w*) was calculated (*σ*^2^*w*), then averaged across all tumors in the cohort. The mean within tumor expression variance was inversly related to the number of biopsies (*k*), denoted as ${{W}}=\scriptstyle\frac{\frac{1}{n}\sum {\sigma }^{2}{{w}}}{k({\mathrm{biopsies}})}$. The total variance (*T*) per gene expression signature was summarized as the sum of mean variance within tumor (*W*) and the variance between tumors (*B* = *σ*^2^*b*). The *W*-to-*T* ratio (*W*/*T*) measures the ITH per signature, with *k* equal to one to ten biopsies investigated in this analysis.

### Survival analyses

OS was used as the primary outcome for prospective validation of survival association. It is defined as the time from registration to death or censoring. Lung-cancer-specific survival was used to measure the time from registration to death caused by lung cancer. DFS is defined as the time from registration to radiologically confirmed recurrence of the primary tumor or death or censoring. Intrathoracic relapses (*n* = 24), extrathoracic relapses (*n* = 14) or both (*n* = 16) were included in our dataset. Two patients with LUAD (CRUK0511 and CRUK0512) involved in the analysis for time to relapse were censored at the time of the diagnosis of new primary cancer owing to uncertainty of whether the subsequent recurrence was from the first primary or the new primary cancer. For patients with multiple synchronous primary LUAD tumors, the average value of genetic metrics was calculated. The HR and *P* value adjusted for age, sex, smoking pack-years, adjuvant treatment, tumor stage (TNM 8th edition) and histologic grade in multivariable Cox regression analyses, and log-rank *P* value between group comparisons were calculated using the survival R package (version 3.5). Kaplan–Meier curves were plotted using the survminer R package (version 0.4.9), whereas the results of multivariable Cox regression analyses were plotted using the forestplot R package (version 3.1.3). All survival analyses were performed on patients with all data available.

### Meta-analysis of ORACLE prognostic values in microarray cohorts of patients with stage I LUAD

Microarray and clinical data were downloaded from GSE50081, GSE31210, GSE30219 and GSE68465 for a total of 580 patients with stage I LUAD enrolled in Shedden et al.^7^, Der et al.^27^, Okayama et al.^28^ and Rousseaux et al.^29^ cohorts. The prognostic value of the ORACLE risk score was tested across four cohorts using the coxph function in the survival package (version 3.5). In the Der et al., Okayama et al. and Rousseaux et al. cohorts, 22 out of 23 genes were available, and in the Shedden et al. cohort, 19 out of 23 genes were available for analysis. The meta-analysis was performed using the rmeta R package (version 3.0).

### Preinvasive lung squamous cell carcinogenesis dataset

Gene expression data published by Mascaux et al.^30^ were downloaded from the Gene Expression Omnibus for 77 patients with lung squamous carcinogenesis (GSE33479). Eight histological stages were identified by the authors, including 27 normal tissues, 15 hyperplasia, 15 metaplasia, 13 mild dysplasia, 13 moderate dysplasia, 12 severe dysplasia, 13 CIS and 14 SCC. This was further summarized as four molecular steps of progression according to the authors, that is, (1) normal and hyperplasia tissues, (2) low-grade lesions including progression from metaplasia to moderate dysplasia, (3) high-grade lesions comprising severe dysplasia and CIS, and (4) the formation of SCC. A linear mixed-effects model was performed using the ORACLE risk score as the response variable and samples as the fixed effect, setting each patient as the random effect. No correction was made for multiple comparisons among developmental stages.

### ORACLE risk score compared between seeding and nonseeding regions

The ORACLE risk score was calculated for each primary tumor region and compared between seeding and nonseeding regions by a linear mixed-effects model setting each tumor as a random effect. Seeding regions were defined as primary tumor regions that contain a most recent shared clone between the primary tumor and metastasis^31^.

### In vitro drug sensitivity screening

The ORACLE risk score was calculated using expression data for cancer cell lines provided in DepMap (version 22Q1), subsetting for LUAD cell lines for subsequent analyses. Drug sensitivity (IC_50_) data were derived from the GDSC database for 396 compounds and 54 LUAD cell lines (Cancer Cell Line Encyclopedia)^34,35^. We filtered out cell lines with data for fewer than 50 compounds and removed compounds with data missing for more than 5 cell lines, leaving 37 cell lines and 359 compounds for subsequent analysis (Extended Data Fig. 6a). To determine the model for assessing association between drug sensitivity and ORACLE, we examined the distribution of IC_50_ values, resulting in nonnormal distributions. Therefore, a Spearman correlation test was applied to the IC_50_ and ORACLE risk score to determine significance (*P* < 0.05) for each drug across the cell lines. No correction was made for multiple comparisons. A list of drugs approved by the FDA for NSCLC was obtained from the National Cancer Institute (https://www.cancer.gov/about-cancer/treatment/drugs/lung). The targeting pathway was derived from the GDSC annotation.

### Determinants for ORACLE magnitude

ORACLE magnitude was defined as the mean risk score among regions for a given tumor. To identify the associated determinants, multiple linear regression models were applied separately for clinicopathological and genetic features in the TRACERx exploratory cohort. Clinicopathological features include patient age, sex, the number of tumor biopsies, tumor stage (TNM version 8), smoking status, tumor volume and Ki67 score. Genetic features including WGD events, FLOH and tumor evolutionary metrics (SCNA-ITH, clonal and subclonal mutations in driver genes, and recent subclonal expansion) were identified in the TRACERx study^14^.

### Clinical outcome variance explained by TRACERx biomarkers

To investigate how much variance of clinical outcome was explained by TRACERx biomarkers including SCNA-ITH, WGD, recent subclonal expansion, detection of preoperative ctDNA, STAS and ORACLE, we applied a generalized linear model treating the survival status at a given follow-up year as a response variable. Within the chosen follow-up time, patients with censored status were removed, keeping patients who had either a death event or no event. The variance explained was calculated using the PseudoR2 function in the DescTools R package (version 0.99.51).

### Enrichment of somatic mutation in NSCLC driver genes

A list of SNVs in driver genes for NSCLC was collated in the TRACERx study^14^. For each SNV at the gene level, the enrichment was calculated using the frequency of mutations and was compared using a two-sided Fisher’s exact test at regional level. The OR was taken at the natural log scale. No correction was made for the multiple comparisons in this analysis.

### Identification of recurrent SCNAs

The genomic regions that represented a recurrent SCNA were identified using GISTIC2.0 (version 2.0.23)^38^. The copy number of a chromosomal segment was normalized against the sample mean ploidy and taken as the input for GISTIC2.0 to identify genomic regions with recurrent amplification or deletion. Amplification and deletion were defined as normalized copy number >log_2_(2.5/2) and <log_2_(1.5/2), respectively. For a given genomic region, the SCNA positive-selection score (G score) was obtained separately for patient cohorts with ORACLE low-risk and high-risk tumors; then, a G-score difference was calculated between the cohorts. A positive G-score difference (>0) with *q* value <0.05 indicated a statistically significant positive selection at the loci.

### Statistical analysis

All statistical tests were performed using R (version 4.3.2). Tests involving correlation were performed using cor.test with the Pearson or Spearman method. Tests involving the comparisons of distributions were performed using wilcox.test with a two-sided Wilcoxon rank-sum test or using the lme function in the nlme R package (version 3.1) with a linear mixed-effects regression analysis. Fisher’s exact tests using fisher.test or chi-squared test using chisq.test were applied to count data to compare frequencies. HRs and *P* values for ORACLE adjusted for clinicopathological factors were calculated using multivariable Cox proportional hazards models. Two-sided log-rank tests were performed for the comparisons between groups in the Kaplan–Meier curves. For all analyses, the number of data points included was plotted or annotated in the corresponding figures and all statistical tests were two-sided unless otherwise specified. *P* < 0.05 was considered as statistically significant unless otherwise specified. The R packages tidyverse (version 2.0.0) and readxl (version 1.4.3) were used for data handling. The plotting was performed using ggplot2 (version 3.5.1), ggalluvial (version 0.12.5), ggrepel (version 0.9.4), ComplexHeatmap (version 2.18.0), pheatmap (version 1.0.12), cowplot (version 1.1.1), gridExtra (version 2.3), scales (version 1.3.0), RColorBrewer (version 1.1), viridis (version 0.6.4), circlize (version 0.4.15), wesanderson (version 0.3.7) and colorspace (version 2.1).

### Statistics and reproducibility

No statistical method was used to predetermine sample sizes of the validation and exploratory cohorts. All available samples that passed the quality-check filters of sequencing data were included in our analyses. Data collection and analysis were not performed blind to the conditions of the study. Our study did not include group assignments and, thus, randomization is not applicable. Data distribution was assumed to be normal, but this was not formally tested. Further information on research design is available in the Nature Research Reporting Summary linked to this article.

### Reporting summary

Further information on research design is available in the Nature Portfolio Reporting Summary linked to this article.

## Supplementary information

Reporting Summary

REMARK checklist.

Supplementary Table 1 Published RNA prognostic signatures. Gene lists from six published signatures for LUAD.

## Source data

Source Data Fig. 1 Statistical source data for Fig. 1.

Source Data Fig. 2 Statistical source data for Fig. 2.

Source Data Fig. 3 Statistical source data for Fig. 3.

Source Data Fig. 4 Statistical source data for Fig. 4.

Source Data Fig. 5 Statistical source data for Fig. 5.

Source Data Extended Data Fig. 1 Statistical source data for Extended Data Fig. 1.

Source Data Extended Data Fig. 2 Statistical source data for Extended Data Fig. 2.

Source Data Extended Data Fig. 3 Statistical source data for Extended Data Fig. 3.

Source Data Extended Data Fig. 4 Statistical source data for Extended Data Fig. 4.

Source Data Extended Data Fig. 5 Statistical source data for Extended Data Fig. 5.

Source Data Extended Data Fig. 6 Statistical source data for Extended Data Fig. 6.

Source Data Extended Data Fig. 7 Statistical source data for Extended Data Fig. 7.

Source Data Extended Data Fig. 8 Statistical source data for Extended Data Fig. 8.

Source Data Extended Data Fig. 9 Statistical source data for Extended Data Fig. 9.

## Data Availability

The RNA-seq data (in each case from the TRACERx study) used during this study have been deposited at the European Genome–phenome Archive, which is hosted by the European Bioinformatics Institute and the Centre for Genomic Regulation, under accession code EGAS00001006517. Access is controlled by the TRACERx data access committee. Details on how to apply for access are available at the linked page. Previously published preinvasive lesion data are available under accession code GSE33479. Four microarray cohorts used for survival validation of ORACLE were available under accession codes GSE68465, GSE50081, GSE31210 and GSE30219. Source data are provided with this paper.

## Code availability

No new code was developed in our study. Codes for processing data and generating figures are available via GitHub at https://github.com/dhruvabiswas/tracerx-oracle2.
