# Supplementary material for: Prospective validation of ORACLE, a clonal expression biomarker associated with survival of patients with lung adenocarcinoma
Source: Nat Cancer. 2025 Jan 9;6(1):86–101. doi: 10.1038/s43018-024-00883-1 (PMC11779643; doi:10.1038/s43018-024-00883-1)
Supplement: Supplementary file 1 — Reporting Summary [file 43018_2024_883_MOESM1_ESM.pdf]

nature portfolio

Corresponding author(s): Dhruva Biswas, Nicolai Birkbak, Charles Swanton

Last updated by author(s): Sep 27, 2024

Reporting Summary

Nature Portfolio wishes to improve the reproducibility of the work that we publish. This form provides structure for consistency and transparency in reporting. For further information on Nature Portfolio policies, see our Editorial Policies and the Editorial Policy Checklist.

Statistics

For all statistical analyses, confirm that the following items are present in the figure legend, table legend, main text, or Methods section.

n/a	Confirmed	
☐	☒	The exact sample size (*n*) for each experimental group/condition, given as a discrete number and unit of measurement
☐	☒	A statement on whether measurements were taken from distinct samples or whether the same sample was measured repeatedly
☐	☒	The statistical test(s) used AND whether they are one- or two-sided<br>*Only common tests should be described solely by name; describe more complex techniques in the Methods section.*
☐	☒	A description of all covariates tested
☐	☒	A description of any assumptions or corrections, such as tests of normality and adjustment for multiple comparisons
☐	☒	A full description of the statistical parameters including central tendency (e.g. means) or other basic estimates (e.g. regression coefficient) AND variation (e.g. standard deviation) or associated estimates of uncertainty (e.g. confidence intervals)
☐	☒	For null hypothesis testing, the test statistic (e.g. *F*, *t*, *r*) with confidence intervals, effect sizes, degrees of freedom and *P* value noted<br>*Give P values as exact values whenever suitable.*
☒	☐	For Bayesian analysis, information on the choice of priors and Markov chain Monte Carlo settings
☒	☐	For hierarchical and complex designs, identification of the appropriate level for tests and full reporting of outcomes
☐	☒	Estimates of effect sizes (e.g. Cohen's *d*, Pearson's *r*), indicating how they were calculated

Our web collection on statistics for biologists contains articles on many of the points above.

Software and code

Policy information about availability of computer code

Data collection

No custom code and software was used for data collection. Codes for processing data and generating figures are available at https://github.com/dhruvabiswas/tracerx-oracle2.

Data analysis

All analyses were performed using R (version 4.3.2) with the following open source packages:

RSEM package version 1.3.3
DESeq2 version 1.42.0
survival version 3.5
survminer version 0.4.9
forestplot version 3.1.3
rmeta version 3.0
DescTools version 0.99.51
GISTIC2.0 version 2.0.23
nlme version 3.1
tidyverse version 2.0.0
readxl version 1.4.3
ggplot2 version 3.5.1
ggalluvial version 0.12.5
ggrepel version 0.9.4
ComplexHeatmap version 2.18.0
pheatmap version 1.0.12

cowplot version 1.1.1
gridExtra version 2.3
scales version 1.3.0
RColorBrewer version 1.1
viridis version 0.6.4
circlize version 0.4.15
wesanderson version 0.3.7
colorspace version 2.1

For manuscripts utilizing custom algorithms or software that are central to the research but not yet described in published literature, software must be made available to editors and reviewers. We strongly encourage code deposition in a community repository (e.g. GitHub). See the Nature Portfolio guidelines for submitting code & software for further information.

Data

Policy information about availability of data

All manuscripts must include a data availability statement. This statement should provide the following information, where applicable:

- Accession codes, unique identifiers, or web links for publicly available datasets
- A description of any restrictions on data availability
- For clinical datasets or third party data, please ensure that the statement adheres to our policy

The RNA-seq data (in each case from the TRACERx study) used during this study have been deposited at the European Genome–phenome Archive, which is hosted by the European Bioinformatics Institute and the Centre for Genomic Regulation, under the accession codes EGAS00001006517. Access is controlled by the TRACERx data access committee. Details on how to apply for access are available at the linked page. Previously published preinvasive lesion data are available under accession code GSE33479. Four microarray cohorts used for survival validation of ORACLE were available under accession codes GSE68465, GSE50081, GSE31210, and GSE30219.

Research involving human participants, their data, or biological material

Policy information about studies with human participants or human data. See also policy information about sex, gender (identity/presentation), and sexual orientation and race, ethnicity and racism.

Reporting on sex and gender

Sex and gender were not considered in the study design, the cohort comprised 233 (55%) males and 188 (45%) females and all available individuals were included in each analysis.

Reporting on race, ethnicity, or other socially relevant groupings

No race-based analysis was performed. No socially relevant categorization variables or terms used.

Population characteristics

Only lung adenocarcinoma patients (184 patients) from the TRACERx study were included in the analysis of this study. There were 94 male and 90 female lung adenocarcinoma patients in the TRACERx study, with a median age of 68. The cohort is predominantly early-stage: Ia(45), Ib(38), IIa(8), IIb(42), IIIa(38), IIIb(13). Sixty-three had no adjuvant treatment and 121 had adjuvant therapy.

Please note that the study started recruiting patients in 2016, when TNM version 7 was standard of care. The up-to-date inclusion/exclusion criteria now utilizes TNM version 8.

TRACERx inclusion and exclusion criteria
Inclusion Criteria:
_Written Informed consent
_Patients ≥18 years of age, with early stage I-IIIB disease (according to TNM 8th edition) who are eligible for primary surgery.
_Histopathologically confirmed NSCLC, or a strong suspicion of cancer on lung imaging necessitating surgery (e.g. diagnosis determined from frozen section in theatre)
_Primary surgery in keeping with NICE guidelines planned

_Agreement to be followed up at a TRACERx site
_Performance status 0 or 1
_Minimum tumor diameter at least 15mm to allow for sampling of at least two tumour regions (if 15mm, a high likelihood of nodal involvement on pre-operative imaging required to meet eligibility according to stage, i.e. T1N1-3)
Exclusion Criteria:
_Any other* malignancy diagnosed or relapsed at any time, which is currently being treated (including by hormonal therapy).
_Any other* current malignancy or malignancy diagnosed or relapsed within the past 3 years**.
*Exceptions are: non-melanomatous skin cancer, stage 0 melanoma in situ, and in situ cervical cancer
**An exception will be made for malignancies diagnosed or relapsed more than 2, but less than 3, years ago only if a preoperative biopsy of the lung lesion has confirmed a diagnosis of NSCLC.
_Psychological condition that would preclude informed consent
_Treatment with neo-adjuvant therapy for current lung malignancy deemed necessary
_Post-surgery stage IV
_Known Human Immunodeficiency Virus (HIV), Hepatitis B Virus (HBV), Hepatitis C Virus (HCV) or syphilis infection.
_Sufficient tissue, i.e. a minimum of two tumor regions, is unlikely to be obtained for the study based on pre-operative imaging

Patient ineligibility following registration

_There is insufficient tissue
_The patient is unable to comply with protocol requirements
_There is a change in histology from NSCLC following surgery, or NSCLC is not confirmed during or after surgery.
_Change in staging to IIIC or IV following surgery
_The operative criteria are not met (e.g. incomplete resection with macroscopic residual tumors (R2)). Patients with microscopic residual tumors (R1) are eligible and should remain in the study
_Adjuvant therapy other than platinum-based chemotherapy and/or radiotherapy is administered.

Recruitment

When patients are initially diagnosed with stage I-III lung cancer and then referred for surgical resection, a research nurse identifies them on a clinic/operating list. The patient has an initial eligibility assessment and then provided with written information about the TRACERx study and he/she can ask the research nurse any questions.

Patients have to agree to provide serial blood samples whenever they attend clinic for routine blood sampling, so this represents the only main potential self-selecting bias (i.e. only patients willing to do this would participate). However, it is unclear how this would affect the biomarker analyses. Also, the gender and ethnicity characteristics are in line with patients seen in routine practice.

Inclusion and exclusion criteria are summarised above.
Informed consent for entry into the TRACERx study was mandatory and obtained from every patient.

Ethics oversight

The study was approved by the NRES Committee London with the following details:
Study title: TRAcking non small cell lung Cancer Evolution through therapy (Rx)
REC reference: 13/LO/1546
Protocol number: UCL/12/0279
IRAS project ID: 138871

Note that full information on the approval of the study protocol must also be provided in the manuscript.

Field-specific reporting

Please select the one below that is the best fit for your research. If you are not sure, read the appropriate sections before making your selection.

☒ Life sciences ☐ Behavioural & social sciences ☐ Ecological, evolutionary & environmental sciences

For a reference copy of the document with all sections, see nature.com/documents/nr-reporting-summary-flat.pdf

Life sciences study design

All studies must disclose on these points even when the disclosure is negative.

Sample size

No statistical methods were used to predetermine sample size. The sample size of 184 lung adenocarcinoma patients that passed quality check filters for RNA represents the half-way point of the TRACERx longitudinal study. In total, 158 patients (369 tumour regions), excluding those profiled in previous training study, were included in the validation analysis. 184 patients (450 tumour regions) were included in exploratory analysis.

Data exclusions

Data was excluded only on the basis of:
- Non-elegibility for the TRACERx clinical trial due to failure of the patient's data to comply with the study protocol (see below)
- The sequenced data did not pass our quality check filters

Replication

TRACERx is a prospective longitudinal study. As such, the results shown here are not the result of an experimental set up. This study reflects hypothesis generating analysis.

Randomization

This is not relevant to the study, as samples were split into high- and low-risk groups using prognostic gene expression signatures.

Blinding

Blinding was not relevant to the study, as there were no control and treatment arms involved.

Reporting for specific materials, systems and methods

We require information from authors about some types of materials, experimental systems and methods used in many studies. Here, indicate whether each material, system or method listed is relevant to your study. If you are not sure if a list item applies to your research, read the appropriate section before selecting a response.

Materials & experimental systems

n/a	Involved in the study
☒	☐ Antibodies
☒	☐ Eukaryotic cell lines
☒	☐ Palaeontology and archaeology
☒	☐ Animals and other organisms
☐	☒ Clinical data
☒	☐ Dual use research of concern
☒	☐ Plants

Methods

n/a	Involved in the study
☒	☐ ChIP-seq
☒	☐ Flow cytometry
☒	☐ MRI-based neuroimaging

Clinical data

Policy information about clinical studies
All manuscripts should comply with the ICMJE guidelines for publication of clinical research and a completed CONSORT checklist must be included with all submissions.

Clinical trial registration
TRACERx Lung https://clinicaltrials.gov/ct2/show/NCT01888601, approved by an independent Research Ethics Committee, 13/LO/1546

Study protocol
https://clinicaltrials.gov/study/NCT01888601

Data collection
Clinical and pathological data is collected from patients during study follow up at the time of and immediately after clinic visit - this period is a minimum of five years. Data collection is overseen by the sponsor of the study (Cancer Research UK & UCL Cancer Trials Centre) and takes place in outpatient respiratory, surgical or oncology clinics at hospital sites where the study is approved and are local to the patient across the United Kingdom. Source data files are maintained by the research team and entered electronically on a centralised database called MACRO that is overseen and governed by the Clinical Trial Centre. Recruitment started in 2014 and is still ongoing (in London and Manchester).

Outcomes
The main clinical outcomes are:
Overall survival – measured from the time of study registration to date of death from any cause.
Lung-cancer-specific survival – measured from the time of study registration to death caused by lung cancer.
Disease-free survival (DFS) – measured from the time of study registration to date of first lung recurrence or death from any cause.
Patients who do not have these events are censored at the date last known to be alive (including patients who developed a new primary tumour that has been shown biologically to not be linked to the initial primary lung tumour).

Plants

Seed stocks
Report on the source of all seed stocks or other plant material used. If applicable, state the seed stock centre and catalogue number. If plant specimens were collected from the field, describe the collection location, date and sampling procedures.

Novel plant genotypes
Describe the methods by which all novel plant genotypes were produced. This includes those generated by transgenic approaches, gene editing, chemical/radiation-based mutagenesis and hybridization. For transgenic lines, describe the transformation method, the number of independent lines analyzed and the generation upon which experiments were performed. For gene-edited lines, describe the editor used, the endogenous sequence targeted for editing, the targeting guide RNA sequence (if applicable) and how the editor was applied.

Authentication
Describe any authentication procedures for each seed stock used or novel genotype generated. Describe any experiments used to assess the effect of a mutation and, where applicable, how potential secondary effects (e.g. second site T-DNA insertions, mosiacism, off-target gene editing) were examined.
